# Systematic review and meta-analysis of the effects of combined physical and cognitive training on depression symptoms in adults across clinical populations

**DOI:** 10.3389/fpsyg.2026.1741014

**Published:** 2026-09-24

**Authors:** Piesie A. G. Asuako, Ghazal Naser, Luis K. H. Holzhauer, Thomas Beblo, Claudia Voelcker-Rehage

**Affiliations:** 1 Department of Neuromotor Behavior and Exercise, University of Münster, Münster, Germany; 2 Chair of Sport Analytics, Saarland University, Saarbrücken, Germany; 3 Department of Psychiatry and Psychotherapy, Medical School and University Medical Center OWL, Bielefeld University, Evangelisches Klinikum Bethel, Bielefeld, Germany; 4 Joint Institute for Individualisation in a Changing Environment (JICE), University of Münster and Bielefeld University, Bielefeld, Germany

**Keywords:** adults, cognitive training, combined training, depression, physical exercise, standardized mean difference

## Abstract

**Objectives:**

To compare the effect of combined physical training (PT) and cognitive training (CT) with single-modality interventions, on depression symptoms in adults across clinical populations.

**Methods:**

PubMed, Scopus, Web of Science, SPORTDiscus, and APA PsycINFO were systematically searched for relevant English-language studies up to December 2025. Search terms focused on combined PT and CT compared with PT or CT alone in adults with depression symptoms. Included samples were predominantly heterogeneous clinical populations, particularly individuals with neurological conditions (e.g., stroke, Parkinson’s disease, multiple sclerosis, dementia, and mild cognitive impairment), as well as chronic somatic conditions (e.g., fibromyalgia), alongside a smaller number of non-clinical (healthy) populations. Data were extracted on training interventions, sample characteristics, and depression symptoms. Between-group standardized mean differences were calculated from post-intervention means and standard deviations using random-effects models to compare combined PT and CT with single-modality interventions.

**Results:**

Sixteen studies were identified (*N* = 813). Across the included studies, both combined and single-modality interventions (PT or CT) were associated with reductions in depression symptoms. However, pooled between-group analyses indicated that combined interventions were associated with greater reductions in depression symptoms than single-modality interventions (SMD = −0.40, 95% CI [−0.69, −0.11], *I*^2^ = 69.7%), although moderate between-study heterogeneity was observed. Most comparisons were conducted against PT alone, with only limited evidence for CT-only comparators. Sensitivity analyses restricted to studies (*n* = 13) using validated depression-specific scales showed a smaller effect in the same direction that was no longer statistically significant (SMD = −0.24, 95% CI [−0.51, 0.03], *I*^2^ = 57.2%).

**Conclusion:**

Combined PT and CT may provide greater reductions in depression symptoms than single-modality interventions, particularly PT alone. However, this effect was attenuated and no longer statistically significant when analyses were restricted to depression-specific measures. Given the heterogeneity, variability in outcome measures, limited evidence for CT-only comparisons, and very low certainty of evidence, findings need to be interpreted cautiously.

**Systematic review registration:**

https://www.crd.york.ac.uk/PROSPERO/view/CRD42024583214, Identifier CRD42024583214.

## Introduction

Depression, a prevalent mental health issue, imposes a substantial burden on both affected individuals and society as a whole (WHO, 2022). According to the World Health Organization (WHO), around 280 million individuals globally experience depression, with an estimated prevalence rate of almost 5% (WHO, 2022). Furthermore, it is estimated that by 2030, depressive disorders will become the leading cause of global disease burden, as measured in disability-adjusted life years (DALYs) (Karrouri et al., 2021; Huang et al., 2022; Liu et al., 2024). In ICD-11, a single depressive episode is defined by the presence of at least five of a list of ten diagnostic criteria (Harrison et al., 2021). One of these criteria must be either a depressed mood or a loss of interest or pleasure, each occurring almost every day for a period of at least 2 weeks. Furthermore, other affective, cognitive, behavioral, or physiological symptoms are associated with significant impairments in the functioning of those affected. Up to 90% of depressive patients report neurocognitive deficits (Schwert et al., 2019), such as deficits in attention, memory, processing speed, and executive functioning (Beblo et al., 2011), and a bidirectional association between cognitive deficits and depression symptoms is proposed (Yin et al., 2024). Notably, depression symptoms and related cognitive deficits can also manifest in persons who do not fully fulfill the clinical diagnostic criteria for depression (Bretschneider et al., 2017). Depression symptoms are highly prevalent in various neurological and physical diseases, such as neurodegenerative diseases (Baquero and Martín, 2015), stroke, and cardiovascular disease (Goodwin, 2006). The symptoms can significantly impact patients’ treatment adherence (Grenard et al., 2011). Grenard et al. (Grenard et al., 2011) found that patients with chronic diseases who exhibit depression symptoms are 1.76 times more likely to be non-adherent to medication compared to patients without depression. This lack of adherence could ultimately exacerbate the medical illness (Schönenberg et al., 2024). According to the Robert Koch Institute in 2015, one in 10 adults in Germany experiences current depression symptoms (Bretschneider et al., 2017). Similarly, a broader review examining the prevalence of clinically relevant depression symptoms across Europe from 2018 to 2020 reported an overall prevalence of 6.4% (Arias-de la Torre et al., 2023). As a result, depression symptoms have emerged as a substantial public health issue (Lépine and Briley, 2011), underscoring the need for increased awareness and management of such symptoms in patients with comorbid diseases as well as the general population.

Treatment of depression symptoms often involves antidepressant medications, such as selective serotonin reuptake inhibitors and others (Bursky et al., 2022). While antidepressant treatment can double the chance of recovery (from 30 to 65%), it carries risks such as side effects, overdose, and unwanted drug interactions, particularly when administered to patients with other medical illnesses (Baldwin, 1995).

Apart from psychotherapy, physical training (PT) (Noetel et al., 2024; Singh et al., 2023) and cognitive training (CT) (Chan et al., 2020; Kim et al., 2023) have also been shown to improve depression symptoms with limited adverse effects. Prior reviews and meta-analyses have demonstrated that both PT and CT interventions are associated with reductions in depression symptoms, with moderate standardized mean differences (SMD) observed for CT (SMD = −0.51) and PT (SMD = −0.61) (Chan et al., 2020; Silveira et al., 2013). PT induces metabolic and neurochemical changes that enhance brain plasticity and can induce antidepressant effects (Pahlavani, 2023; Li et al., 2024). Specifically, PT has been shown to increase serotonin, dopamine, and GABA levels while reducing inflammatory markers, all of which contribute to improved mood (Pahlavani, 2023). Interestingly, structural and functional brain plasticity have also been shown to be enhanced by PT (Kraft, 2012). CT also targets interconnected brain networks responsible for cognition and emotion and might address depression by enhancing impaired cognitive functions (Chan et al., 2020; Wang et al., 2016).

Based on the individual beneficial impact of PT and CT, combined PT and CT interventions have been developed to maximize training efficiency (Bamidis et al., 2015). Numerous reviews have consistently demonstrated that combined PT and CT interventions, such as dancing, Taichi, exergaming as interactive digital, augmented, or virtual game-like environments, or multimodal training, yield equal or greater benefits on cognitive performance (e.g., executive functions, speed, and global cognition) in adults across various cognitive states than each intervention alone (Stojan and Voelcker-Rehage, 2019; Lauenroth et al., 2016). The additive and stronger effect of the combined training might be attributed to the inherent connection between PT and CT processes, mediated through reciprocal stimulation of neuroplasticity (Heisz et al., 2017). As a result, combined PT and CT interventions hold promise in maximizing the reduction of depression symptoms, given the significant association between cognitive functioning (Kim and Jung, 2018), neuroplasticity (Yeh et al., 2023), and depression symptoms.

However, limited review articles (Huang et al., 2022; Dhir et al., 2021) have explored the effect of combined PT and CT on depression symptoms compared to PT or CT alone: Dhir et al. (2021) investigated the effects of combined and CT interventions on enhancing inhibitory control—a cognitive dimension that is associated with depression symptoms (Nahum et al., 2023)—in adolescents, young adults, and older adults. They reported small yet significant positive effects across all studies. Huang et al. (2022) reviewed the effect of exergaming, as a form of combined PT and CT, on depression symptoms in the adult population and concluded that exergaming reduced depression symptoms to a larger extent than an active or passive control condition. Unfortunately, neither study exclusively compared combined training with single PT or CT. This missing comparison complicates conclusions about whether observed effects on depression symptoms are attributable to the synergistic integration of both interventions or the impact of either alone.

To the best of our knowledge, a comprehensive exploration and synthesis of the effects of combined PT and CT interventions on depression symptoms of adults versus either PT or CT alone has not been evaluated and summarized in the context of a systematic review and meta-analysis. Therefore, the objectives of this systematic review were to gather evidence on whether there are greater effects of combined PT and CT interventions on depression symptoms in adults across clinical populations suffering from depression or revealing depression symptoms as side effects of their disease versus single PT or CT. We hypothesized that combined PT and CT interventions have a greater impact on depression symptoms than either intervention alone. By examining the available evidence, our review aimed to provide a more holistic understanding of the effectiveness of this combined strategy in alleviating depression symptoms, offering efficient methods for both the general population and patients with other illnesses. We also aimed to shed light on the moderators and biological mechanisms that may underlie their effects.

## Methods

### Registration

The systematic review follows the Preferred Reporting Items for Systematic Reviews (PRISMA; www.prisma-statement.org) guidelines (Page et al., 2021). This approach guarantees thorough and transparent reporting of the methods and results. Further details of the PRISMA checklist are provided (cf. Supplementary materials, Table 1). The study protocol was prospectively registered in PROSPERO (International Prospective Register of Systematic Reviews; CRD42024583214), maintained by the National Institute for Health and Care Research, and was followed with minor deviations, including database expansion and an updated search timeline. To assess robustness, we conducted subgroup analyses by clinical characteristics, depression scale, and intervention type, as well as a sensitivity analysis, to examine their influence on the pooled SMD and statistical heterogeneity. As the protocol had planned only qualitative exploration of effect modifiers, these analyses constitute a deviation and should be interpreted as exploratory.

### Search strategy

A systematic electronic database search was conducted to identify relevant studies. Five databases–PubMed, Scopus, Web of Science, SPORTDiscus and APA PsycINFO–were systematically searched to identify relevant studies up to December 2025, with no specific limitations on publication dates. The search terms were intersections of terms referring to the combination of PT and CT in adults with depression symptoms. Furthermore, the reference lists of included studies and studies included in previous reviews were manually searched to identify potentially eligible studies. The full search strategy for each database, including the filters used, is presented in Supplementary materials (cf. Supplementary materials, S1).

### Eligibility criteria

We defined the study eligibility to include peer-reviewed studies only published in English-language involving adults (≥18 years) in whom depressive symptoms or depression-related outcomes were assessed, as evidenced by validated depression assessment scales (e.g., Geriatric Depression Scale (GDS), Hospital Anxiety and Depression Scale (HADS),: Patient Health Questionnaire (PHQ), Beck Depression Inventory (BDI), Cornell Scale for Depression in Dementia(CSDD)), or non-specific (related) measures that include depression component (e.g., European Quality of Life-5 Dimensions (EQ-5D), Fibromyalgia Impact Questionnaire(FIQ), reported as mean (M) scores with corresponding standard deviations (SD). The inclusion criteria were primarily determined using the Population, Intervention, Comparator, Outcome, and Study Design (PICOS) framework. To ensure a comprehensive synthesis of the evidence, the population was not restricted by setting (clinical or community) or comorbid health status (e.g., stroke, dementia). To address clinical heterogeneity, subgroup analyses by clinical characteristics were conducted (see Data Synthesis and Statistical Analysis): (a) Participants: Adults aged 18 years or older with depression symptoms as measured by any validated depression scale or related scales (M_depression score_ ± SD) were included. Participants were included regardless of disease status or setting (e.g., clinical or community). (b) Intervention: Combined PT and CT interventions were included. PT was defined according to the American College of Sports Medicine as any bodily movement done to improve or maintain one or more components of physical fitness (Nelson et al., 2007). CT was defined as drill and practice or compensation strategies for a particular cognitive domain, such as executive functions (Chan et al., 2020). Combined PT and CT interventions were categorized as: (a) simultaneous-incorporated training: PT and CT integrated into a single, cohesive activity where cognitive task is embedded in the physical movement (e.g., exergaming or virtual reality video games) (Manser et al., 2023; Weike et al., 2026), (b) simultaneous (independent) training, also known as dual-task training: PT and CT performed at the same time but as unrelated tasks (e.g., stationary cycling while solving math problems) (Chuang et al., 2023; Tait et al., 2017), and (c) sequential training: PT and CT performed in separate, consecutive sessions (Chuang et al., 2023). We included only studies that investigated the effects of combined PT and CT intervention without additional interventional components (e.g., additional CT or PT, or dietary), unless a similar additional CT or PT was applied to the control group, ensuring that the combined training was the only differentiating variable. (c) Comparison: The intervention in the control group consisted of either CT alone or PT alone. In line with the Cochrane Handbook for Systematic Reviews of Interventions, we planned to pool multiple control arms within studies where applicable to avoid unit-of-analysis errors; however, no included study contained both PT-only and CT-only control groups. Treatment as usual was accepted only if a CT or PT was administered alongside. (d) Outcome: Depression scores at baseline and immediately at the end of the intervention, as measured by a validated scale, including self-report or observer-based rating questionnaires and interviews, were considered. (e) Study Design: Randomized controlled trials (RCTs) and cluster-RCTs were included in this review. More details are shown in the Supplementary materials (cf. Supplementary materials, Table 2).

### Study selection

Three independent, blinded reviewers conducted the study selection process. Duplicate studies were removed. Reviewers independently screened the retrieved records based on titles and abstracts, using the inclusion criteria to identify potentially eligible studies. This screening process was performed using Rayyan software (https://new.rayyan.ai/). The reviewers conducted the screening manually within the software. Subsequently, the same reviewers assessed the full texts of potentially eligible studies. Any discrepancies or conflicts were resolved through a consensus discussion involving a fourth reviewer who was not directly involved in the screening process. Studies meeting the inclusion criteria were then included in the review and prepared for data extraction.

### Synthesis methods

After scanning the included studies, three independent reviewers systematically extracted information, blinded and manually, based on our predetermined main study characteristics and entered it into an Excel table. This included details such as the first author, country, sample characteristics (number of participants, distribution of females, M_age_ with SD, age range, and health status) for each group, study design, and group size. Additionally, intervention details were documented, adhering to the components of the F.I.T.T. principles (Katsukawa, 2016), including exercise frequency per week, intensity, duration of total intervention, exercise type for each group, and delivery mode (a group session or individual training). We extracted the targeted cognitive domain of the combined intervention, if mentioned. Finally, we extracted the depression measurement tool used and the results of assessments immediately after the intervention, including within-group changes, and we assessed risk of bias. Furthermore, data from all control groups were thoroughly extracted. Table 1 presents a comprehensive review of the characteristics of each study.

**Table 1:** Study characteristics and risk of bias evaluation for the included studies.

Author/country	Sample (*n*, Age (M, SD))	Design	Intervention	Intervention type	Exercise intensity	Cognitive dimension	Measure of depression	Results	Risk of bias
Ayari et al. (2023), France	EG: 11, age = 79.8y (7.7), *f* = 9, CG: *n* = 12, age = 77.2 y (5.3), *f* = 7, age range: 65–99 y disease: MCI	Single-blinded RCT, two groups	16 weeks, 1 × 60 min/week EG: improvised dancing to music, delivery mode: group; CG: aerobic exercises, delivery mode: groups	Simul. –inc. training	60–70% HRR	Attention	GDS	EG ↓ in GDS CG ↓ in GDS	Some concerns
Baek et al. (2024), South Korea	EG: 22, age: n.a. *f* = 15, CG: *n* = 22, age: n.a. *f* = 14, age range: 65–99 y disease: MCI	Single-blinded RCT, two groups	6 weeks, 3 × 40 min/week EG: dual-task resistance exercise, delivery mode: group; CG: resistance exercise, delivery mode: group	Simul. training	1RM	Dual tasks	GDS	EG ↓ in GDS CG ↓ in GDS	Some concerns
Bisbe et al. (2020), Spain	EG:18 pre/17 post, age = 72.8 y (5.6), *f* = 8 CG: 18 pre/17 post, age = 77.2y (5.1), *f* = 7, age range: 65–85 y disease: MCI	double-blinded RCT, two groups	12 weeks, 2 × 60 min/week EG: choreographed aerobic dance, delivery mode: group; CG: multimodal physical exercises, delivery mode: group	Simul. –inc. training	RPE	Visual memory	HADS	EG, CG no sig. Change in HADS	Low
Carvalho et al. (2020), Brazil	EG: 16 pre/11 post, age = 55.6 y (9.1), *f* = n.a CG: 19 pre/10 post, age = 47.7 y (15.4), *f* = n.a, age range: 18–99 y disease: fibromyalgia	Single blinded RCT, two groups	7 weeks, 3 × 60 min per week EG: exergames (Nintendo) delivery mode: individualized CG: stretching delivery mode: group	Simul. –inc. training	n.a.	n.a.	FIQ	EG ↓ in FIQ CG: no sig. Change in FIQ	Some concerns
de Rooij et al. (2021), Netherlands	EG: 28, age = 65.3 y (9.8), *f* = 10; CG: 27 pre/24 post, age = 61.6 y (10.3), *f* = 6, age range: 18–80 y disease: stroke	single-blinded RCT, two groups	6 weeks, 2 × 30 min per week EG: treadmill walking with VR, delivery mode: individualized; CG: 10–15 min treadmill walking + 15 min functional gait exercises delivery mode: individualized	Simul. –inc. training	EG: RPE; treadmill speed, number of simultaneous tasks; CG: treadmill speed progressive	dual tasks	HADS	EG, CG: no sig. Change in HADS	Low
Ferraz et al. (2018), Brazil	EG: 22 pre/20 post, age = 67 y (1.5), *f* = 10, CG1: 25 pre/22 post, age = 67 y (5.2), *f* = 15, CG2: 25 pre/20 post, age = 71 y (6.7), age range: 60–99 y disease: Parkinson’s disease	Single-blinded pilot RCT, three groups	8 weeks, 3 × 50 min per week EG: exergames (Kinect), delivery mode: individual; CG1: functional training, delivery mode: individualized;CG2: cycling, delivery mode: individualized	Simul. –inc. training	EG: progressive; CG1: progressive; CG2: 50–75% HRmax, progressive	Executive functions	GDS-15	EG, CG1, CG2: no sig changes in GDS-15	Low
Galperin et al. (2023) Israel, USA, Germany	EG: 60 pre/56 post, age = 49.0 y (10.0), *f* = 43, CG: 64 pre/54 post, age = 49.1 y (9.7) *f* = 42, age range: 20–65 y disease: multiple sclerosis	Single-blinded RCT, two groups	6 weeks, 3 × 60 min per weekEG: treadmill walking with VR, delivery mode: individualized; CG: treadmill training,delivery mode: individualized	Simul. –inc. training	Treadmill speed	processing speed	PHQ	EG ↓ in PHQ CG: no sig. Change in PHQ	Low
Khushnood et al. (2021), Pakistan	EG: 45 pre/42 post, age = n.a., *f* = 16; CG: 45 pre/41 post, age = n.a., *f* = 16, age range: 60–99 y healthy	double-blinded RCT, two groups	8 weeks, 2 × 30 min per week EG: exergaming (Nintendo), delivery mode: individualized; CG: balance training, delivery mode: individualized	Simul. –inc. training	n.a.	n.a.	EQ-5D	EG ↓ in EQ-5D-5L CG ↓ in EQ-5D-5L	Some concerns
Lin et al. (2020), Taiwan	EG: 38, age = 64.5 y (13.5), *f* = 11, CG: 114 pre/107 post, age = 66.9 y (13.3), *f* = 47, age range: 20–99 y disease: stroke	Single-blinded RCT, two groups	8 weeks, 5 × 60 min per week EG: early rehabilitation + VR training of 15 min twice a day for 5 days, delivery mode: individualized; CG: early rehabilitation, delivery mode: n.a	Simul. –inc. training	EG: speed, frequency. CG: n.a.	Math calculations, color discrimination	HADS	EG ↓ in HADS CG: no sig changes in HADS	Low
Ozdogar et al. (2020), Turkey	EG: 21 pre/20 post, age = 39.2 y (8.6), *f* = 16; CG1: 19 pre/17 post, age = 43.6 y (10.5), *f* = 12, CG2: 20, age = 37.9 y (12.4), *f* = 15, age range: 18–99 y disease: multiple sclerosis	Single-blinded RCT, three groups	8 weeks, 1 × 45 min per week EG: exergame (Kinect), delivery mode: individualized; CG1: conventional rehabilitation delivery mode: n.a CG2: wait-list	Simul. –inc. training	EG: progressive game difficulty CG: reps, sets numbers	Working memory	BDI	EG ↓ in BDICG1, CG2:no sig. Change in BDI	Some concerns
Ozdogar et al. (2022), Turkey	EG: 15 pre/12 post, age = 37.5 y (10.4), *f* = 11; CG: *n* = 15 pre/14 post, age = 37.7 y (13.1), *f* = 10; age range: 18–99 y, disease: multiple sclerosiss	Single-blinded pilot RCT, two groups	45 min/day during the hospitalization period EG: exergame (Kinect); delivery mode: individualized CG: conventional Rehabilitation delivery mode: individualized	Simul. –inc. training	EG: Reps, sets number; CG: Reps, sets, number	Working memory	HADS	EG, CG: no sig. Change in HADS	Some concerns
Song and Park (2015), South Korea	EG: *n* = 20, age = 51.37 y (40.6), *f* = 10, CG: *n* = 20, age = 50.10 y (7.83), *f* = 8; age range: n.a. disease: stroke	Non-blinded RCT, two groups	8 weeks, 5 × 30 min per week EG: exergaming (Kinect), delivery mode: individualized; CG: stationary cycling, delivery mode: individualized	Simul. –inc. training	EG: n.a. CG: 40% HR	n.a.	BDI	EG ↓ in BDI CG ↓ in BDI	Some concerns
Swinnen et al. (2024), Belgium	EG: 9 pre/7 post; age = 81.9 y (8.2), *f* = 7, CG: 14 pre/11 post, age = 84.2 y (5.9), *f* = 10, age range: 60–99 y, disease: MCI	Non-blinded pilot RCT, two groups	12 weeks, 3 × 30 min per week, EG: exergame (VITAAL), delivery mode: individualized; CG: 15 min walking and 15 min squatting and stepping, delivery mode: individualized	Simul. –inc. training	EG: n.a. CG: n.a.	short-term memory	CSDD	EG, CG: no sig. Change in CSDD	Some concerns
Tollár et al. (2019), Netherlands	EG: 25, age = 70.0 y (4.6), *f* = 13, CG1: 25, age = 70.6 y (4.1), *f* = 14, CG2: 24, age: 67.5 y (4.2) *f* = 11, age range: ≥ 60 disease: Parkinson’s disease	Single-blinded RCT, three groups	5 weeks, 5 × 60 min per week EG: exergame (Kinect), delivery mode: group; CG1: stationary Cycling CG2: wait-list delivery mode: group	Simul. –inc. training	EG: 80% HRmax CG: RPE	n.a	BDI	EG ↓ in BDI CG1 ↓ in BDI CG2 no sig. Change in BDI	Low
Yoon et al. (2013), South Korea	EG: 15 pre/11 post, age = 77.9 y (7.5), *f* = n.a. CG: 15 pre/9 post, age = 70.1 y (12.2) *f* = n.a.; age range: 60–99 y, disease: dementia	Non-blinded RCT, two groups	12 weeks, 3 × 30 min per week EG: 20 min cycling + 20 min sequential memory training, delivery mode: n.a.; CG: memory training, delivery mode: n.a.	Seq. training	n.a.	Short-term memory	GDS	EG ↓ in GDS CG: no sig. Change in GDS	Some concerns

*Age was expressed as median and interquartile range; BDI: Beck Depression Inventory; CG: control group; CSDD: Cornell Scale for Depression in Dementia; EG: experimental group; EQ-5D-5L: European Quality of Life-5 Dimensions; F: female numbers; FIQ: Fibromyalgia Impact Questionnaire; GDS: Geriatric Depression Scale; GDS-15: 15-item Geriatric Depression Scale; HADS: Hospital Anxiety and Depression Scale; HAMD: Hamilton Depression Rating Scale; MCI: mild cognitive impairment; n.a: not available; PHQ-9: Patient Health Questionnaire-9; RPE: Rating of Perceived Exertion; 1RM: one-repetition maximum; y: years; ↓ significant reduction in within group; Simul. –inc. training: Simultaneous-incorporated training; Simul. training: Simultaneous training; Seq. training: Sequential training.

The outcomes of depression symptom were extracted as continuous variables from validated depression-specific or related questionnaires (e.g., PHQ-9, BDI, BDI-II, FIQ). For each study, baseline and post-intervention means (M), standard deviations (SD), and sample sizes were extracted for the intervention and comparator groups included in the quantitative synthesis. Although some studies included additional wait-list control groups, these were not included in the quantitative analyses because they did not meet the predefined comparator criteria. Where reported, within-group changes and corresponding *p*-values were also extracted (Tables 1, 2). Mean change scores (post-intervention minus pre-intervention) were calculated for descriptive purposes and are presented in Table 2. However, the meta-analysis used post-intervention M scores and corresponding SDs for the intervention and control groups. To ensure comparability across studies using different depression assessment instruments, we used SMDs to pool effect sizes, in accordance with established methodological recommendations when multiple instruments assessed the same underlying construct (Andrade, 2020). The included outcome measures comprised both validated depression-specific instruments (e.g., GDS, HADS, HAM-D, PHQ-9, BDI, BDI-II) and validated multidimensional instruments containing depression-related components (e.g., EQ-5D3, FIQ). For the EQ-5D, the anxiety/depression dimension (1-3), rather than the overall EQ-5D score was extracted, whereas for the FIQ, we extracted the specific depression domain, reported in cm, rather than the FIQ total score. Although these instruments differ in their primary purpose and psychometric characteristics, all contributed information on depression symptoms, or depression-related constructs are relevant to the present review. All outcomes were oriented such that higher scores indicated greater symptom severity. While SMDs standardize outcomes to a common metric, they may not fully account for differences in psychometric properties, such as reliability, sensitivity, or responsiveness, across measurement instruments (Takeshima et al., 2014). Nevertheless, the included scales assess the same clinical construct of depression symptom severity, and differences between instruments primarily reflect measurement characteristics rather than substantively distinct outcomes (Higgins et al., 2016). Accordingly, pooling using SMD was considered appropriate, while acknowledging that residual measurement variability may contribute to between-study heterogeneity. SMDs (Hedges’ g) were calculated from post-intervention mean scores using the pooled SD of the intervention and control groups, with a correction for small sample bias.

$$\mathrm{SMD}=\frac{\left({\bar{X}}_{\text{intervention},\text{post}}-{\bar{X}}_{\text{control},\text{post}}\right)}{SD_{\text{pooled}}}$$

**Table 2:** Descriptive results of depressive scores.

Study	Groups	Pre	Post	*p*	MD	Group*time
Ayari et al. (2023)	EG: PT + CT	6.5 (1.7)	2.5 (1.8)	**<0.001**	−4.00	n.a.
	CG: PT	8.1 (2.6)	4.1 (2.9)	**<0.001**	−4.00	
Baek et al. (2024)	EG: PT + CT	20.2 (1.2)	17.2 (1.2)	**<0.001**	−3.00	0.10
	CG: PT	20.8 (1.5)	18.6 (1.4)	<0.001	−2.20	
Bisbe et al. (2020)	EG: PT + CT	6.5 (3.0)	6.0 (3.4)	0.28	−0.50	n.a.
	CG: PT	6.0 (3.0)	5.3 (3.5)	0.46	−0.70	
Cano-Mañas et al. (2020)	EG: PT + CT	2.0 (0.6)	1.3 (0.05)	**<0.01**	−0.70	n.a.
	CG: PT	2.4 (0.7)	2.2 (0.8)	<0.01	−0.20	
Carvalho et al. (2020)	EG: PT + CT	8.5 (1.9)	5.9 (3.1)	**<0.05**	−2.60	**0.020**
	CG: PT	8.3 (1.8)	8.3 (2.3)	>0.05	0.00	
de Rooij et al. (2021)	EG: PT + CT	4.3 (3.3)	4.0 (3.4)	> 0.05	−0.30	n.a.
	CG: PT	3.5 (2.2)	2.8 (2.1)	>0.05	−0.70	
Ferraz et al. (2018)	EG: PT + CT	5.3 (3.6)	4.9 (4.4)	0.12	−0.40	n.a.
	CG1: PT	5.4 (2.8)	4.6 (4.1)	0.09	−0.80	
	CG2: PT	3.6 (3.2)	3.8 (2.4)	0.96	0.20	
Galperin et al. (2023)	EG: PT + CT	5.0 (6.5)	3.7 (3.9)	**<0.05**	−1.30	n.a.
	CG: PT	4.8 (7.2)	5.5 (6.3)	0.25	0.70	
Khushnood et al. (2021)	EG: PT + CT	2.0 (0.6)	1.3 (0.4)	**<0.001**	−0.70	n.a.
	CG: PT	2.2 (0.4)	1.7 (0.5)	**<0.001**	−0.50	
Lin et al. (2020)	EG: PT + CT	12.1 (2.5)	9.3 (3.2)	**<0.001**	−2.80	**0.005**
	CG: PT	10.3 (4.8)	10.0 (4.5)	0.69	−0.30	
Ozdogar et al. (2020)	EG: PT + CT	13.4 (6.0)	9.6 (6.0)	**<0.05**	−3.80	n.a.
	CG1: PT	13.9 (9.8)	12.1 (10.1)	0.14	−1.80	
	CG2: wait-list	9.6 (8.0)	8.7 (5.9)	0.41	−0.85	
Ozdogar et al. (2022)	EG: PT + CT	6.9 (4.4)	5.6 (5.8)	>0.05	−1.30	0.960
	CG: PT	6.3 (4.1)	5.6 (5.0)	>0.05	−0.70	
Song and Park (2015)	EG: PT + CT	21.2 (3.8)	14.1 (2.4)	**<0.05**	−7.10	n.a.
	CG: PT	19.6 (3.2)	17.5 (2.7)	**<0.05**	−2.10	
Swinnen et al. (2024)	EG: PT + CT	6.7 (3.0)	5.9 (2.3)	0.34	−0.80	n.a.
	CG: PT	7.0 (4.9)	6.9 (4.7)	0.72	−0.10	
Tollár et al. (2019)	EG: PT + CT	12.4 (2.7)	9.9 (2.2)	**<0.001**	−2.50	n.a.
	CG1: PT	12.7 (3.2)	10.6 (2.3)	**<0.001**	−2.10	
	CG2: wait-list	12.4 (2.9)	12.5 (3.5)	>0.05	0.1	0.001*
Yoon et al. (2013)	EG: PT + CT CG: CT	12.2 (0.9) 11.2 (1.1)	10.6 (1.0) 10.9 (1.3)	**<0.05** >0.05	−1.60 −0.30	n.a.

Pre- and Post-Intervention for the Experimental and Control Groups: Within-Group *p*-VALUES, Mean Differences (MD), and Interaction Effects. Values are displayed by M(SD); EG, experimental group; CG1, first control group; CG2, second control group; n.a., not available; values in bold were statistically significant; *: interaction effect EG, CG1 versus CG2.

### Data synthesis and statistical analysis

The meta-analysis was conducted using R version 4.5.1 with the meta package (v4.17–0) (Balduzzi et al., 2019). SMDs (Hedges’ *g*) with 95% CIs were calculated from post-intervention mean scores by standardizing the difference between the intervention and control group means using the pooled post-intervention SD. Using conventional definitions, SMD estimates of 0.20, 0.50, and 0.80 were considered small, medium, and large, respectively (Andrade, 2020). A negative SMD indicated that the combined PT and CT groups decreased depression symptoms scores more than the control groups. When studies reported more than one eligible comparator group, comparator groups were combined to create a single pairwise comparison, thereby avoiding double-counting of participants and unit-of-analysis errors. Combined sample sizes, means, and standard deviations were calculated according to the procedures recommended in the Cochrane Handbook for Systematic Reviews of Interventions (Cumpston et al., 2019). For studies reporting continuous outcomes as medians and interquartile ranges rather than *M* and *SD*, the reported sample sizes were incorporated into a changing-weight estimator to derive optimal estimates of the *M* and *SD* (Luo et al., 2018; Wan et al., 2014). If summary statistics (e.g., M or SD) were unavailable, the authors were contacted twice to request the missing information. Where data remained unavailable, analyses were performed using the available data in accordance with Cochrane recommendations. Missing outcome data resulting from participant dropout were considered as part of the risk-of-bias assessment rather than being statistically imputed (Higgins et al., 2016; Borenstein et al., 2021). Heterogeneity was assessed using Cochrane’s Q test and quantified using the *I*^2^ statistic, which represents the percentage of total variation across studies due to between-study heterogeneity rather than sampling error. In accordance with Cochrane recommendations, *I*^2^ values of approximately 25, 50, and 75% were interpreted as representing low, moderate, and high heterogeneity, respectively. To account for the potential clinical and methodological diversity across studies, a random-effects model was pre-specified for the primary analysis, as it provides a more conservative estimate of the pooled effect size by considering both within-study and between-study variance. Furthermore, to explore the potential influence of clinical characteristics, depression scale, and intervention type on the pooled SMD and between-study heterogeneity, subgroup analyses were conducted where sufficient data were available. A sensitivity analysis was also performed to assess whether inclusion of non-dedicated (related) depression measures influenced the overall pooled estimate. These analyses deviate from the pre-registered PROSPERO protocol, which had planned only qualitative exploration of potential effect modifiers, and should therefore be interpreted as exploratory rather than confirmatory (Higgins et al., 2016; Borenstein et al., 2021). Moreover, several subgroup analyses included only a small number of studies and were therefore unlikely to have sufficient statistical power to reliably detect differences between subgroups; consequently, these findings should be regarded as hypothesis-generating. Finally, publication bias and small-study effects were assessed using Funnel plot asymmetry and Egger’s regression test to evaluate whether the observed pooled effect size may have been influenced by selective publication or reporting of studies with statistically significant results. Where appropriate, the trim-and-fill method was applied as a sensitivity analysis to estimate the potential impact of missing studies on the pooled effect size (Egger et al., 1997). This was examined through visual inspection of funnel plots and formally tested using Egger’s regression test. These analyses were performed to enhance the transparency and credibility of the meta-analytic findings and to support cautious interpretation of the results.

### Study risk of bias assessment

Reviewers independently evaluated the methodological quality of the studies included in the review using version 2 of the Cochrane tool for assessing risk of bias in randomized trials (RoB2) (Higgins et al., 2016). The assessment considered five aspects: 1. Bias arising from the randomization process, 2. Bias due to deviations from intended interventions, 3. Bias due to missing outcome data, 4. Bias in measurement of the outcome, 5. Bias in selection of the reported result, and finally overall bias. Each aspect was rated as low, high, or with some concerns.

### Certainty of evidence assessment (GRADE)

The certainty of evidence was independently assessed using the GRADE approach. The assessment considered five domains: (1) risk of bias, (2) inconsistency, (3) indirectness, (4) imprecision, and (5) publication bias. Evidence from randomized controlled trials was initially rated as high certainty and subsequently downgraded based on these domains. We evaluated inconsistency using statistical heterogeneity (*I*^2^) and variability in effect estimates, and assessed imprecision based on confidence intervals and sample size. Indirectness was assessed with respect to population characteristics, interventions, and outcomes. Publication bias was assessed using Funnel plot asymmetry and Egger’s regression test. Each outcome was rated as high, moderate, low, or very low certainty.

## Results

### Study selection

A flow chart (cf. Figure 1) provides an overview of our search and selection process. Our electronic database search identified 6,876 studies across Scopus, PubMed, SPORTDiscus, APA PsycINFO, and Web of Science. After removing duplicates (*n* = 1,040), title and abstract screening (*n* = 5,803 removed), 33 reports were sought for full-text retrieval, of which 2 (Yokoi et al., 2022; Li et al., 2018) could not be obtained. The remaining 31 full-text articles were assessed for eligibility. Of these, 13 studies met our eligibility criteria (*n* = 18 removed: wrong study design = 4) (McDougall et al., 2015; Zhu et al., 2022; Lin et al., 2020; Norouzi et al., 2024), wrong intervention, not addressing CT = 5 (Cetin et al., 2022; Hong et al., 2023; Meldrum et al., 2015; Thompson et al., 2013; Rutkowski et al., 2022), wrong control group = 9 (Jeong et al., 2021; Szczepańska-Gieracha et al., 2021; Merchant et al., 2021; Ozdogar et al., 2023; Seo et al., 2023; Santos et al., 2015; Jóźwik et al., 2021; Vieira et al., 2018; Zhou et al., 2020). In addition to the electronic search, three additional records (Baek et al., 2024; Bisbe et al., 2020; Song and Park, 2015) were identified through manual searching (reference list screening and citation tracking) of included studies and relevant reviews. In total, we included 16 studies in our systematic review and meta-analysis that investigated the effect of combined PT and CT interventions on depression symptoms compared to either modality alone.

**Figure 1:**
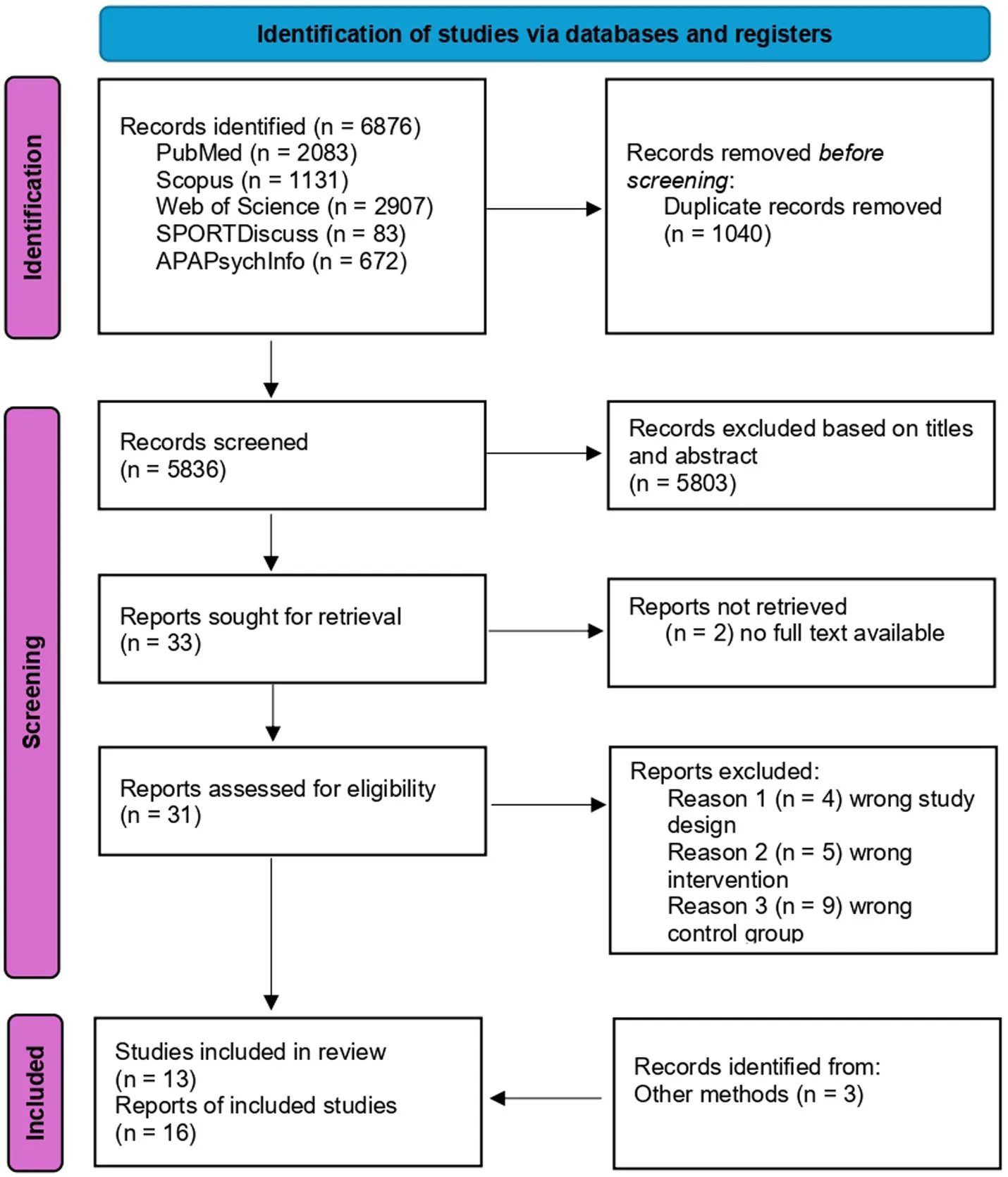
PRISMA flow chart of the search process.

### Study design and participant characteristics

Table 1 summarizes the main characteristics of the included studies. All 16 included studies were published between 2013 and 2024, with most published between 2020 and 2024 (*n* = 13). The studies were conducted in various countries worldwide, including the Netherlands (*n* = 2), South Korea (*n* = 3), Brazil (*n* = 2), Turkey (*n* = 2), Spain (*n* = 2), France (*n* = 1), Pakistan (*n* = 1), Taiwan (*n* = 1), Belgium (*n* = 1), and one study was conducted in three different countries (Israel, USA, and Germany). Thirteen studies were conducted as randomized controlled trials (RCTs) (Baek et al., 2024; Song and Park, 2015; Ayari et al., 2023; Cano-Mañas et al., 2020; de Rooij et al., 2021; Galperin et al., 2023; Khushnood et al., 2021; Ozdogar et al., 2020; Carvalho et al., 2020; Yoon et al., 2013), including two pilot RCTs (Ferraz et al., 2018; Swinnen et al., 2024). Three studies were performed as randomized clinical trials (Bisbe et al., 2020; Ozdogar et al., 2022; Tollár et al., 2019). Thirteen studies included one control group, incorporating either PT (*n* = 12) or CT (*n* = 1), and three studies included two control groups each; in one study, both control groups were active (PT) (Ferraz et al., 2018), while in the other two studies, one control group was active (PT) and the other was passive (Ozdogar et al., 2020; Tollár et al., 2019).

The included samples predominantly comprised heterogeneous clinical populations with neurological and medical comorbidities (e.g., dementia, stroke, Parkinson’s disease, fibromyalgia), rather than individuals with a primary depressive disorder. Participant recruitment took place in senior day care centers, hospitals, rehabilitation centers, and outpatient clinics. Across the 16 included studies, 857 participants were randomized: 363 were allocated to combined PT and CT interventions and 494 to comparator groups (PT alone = 441, CT alone = 9, wait-list = 44). For the quantitative synthesis, only participants from eligible active comparator groups (PT or CT alone) contributed to the meta-analysis. The number of females in the studies was 465 (54.3%), with two studies not reporting the female proportion (Yoon et al., 2013; Tollár et al., 2019). Group sizes ranged from 9 to 152 participants. Based on the reported mean age, 11 studies included predominantly older adults (mean age ≥60 years), while four studies had a mean age below 60 years (Galperin et al., 2023; Ozdogar et al., 2020, 2022; Carvalho et al., 2020). Song and Park (Song and Park, 2015) were considered separately because the study did not clearly report age eligibility criteria. Across the included studies, reported mean ages ranged from approximately 37 to 82 years. Most studies included clinical populations including adults with fibromyalgia in one study (Carvalho et al., 2020), mild cognitive impairment in four studies (Baek et al., 2024; Bisbe et al., 2020; Ayari et al., 2023; Swinnen et al., 2024); seven studies involved participants with neurodegenerative disorders, including Parkinson’s disease (Ferraz et al., 2018; Tollár et al., 2019), dementia (Yoon et al., 2013), multiple sclerosis (Galperin et al., 2023; Ozdogar et al., 2020, 2022), while four studies included adults with stroke (Song and Park, 2015; Cano-Mañas et al., 2020; de Rooij et al., 2021; Lin et al., 2020). One study included participants having no serious systemic disease (Khushnood et al., 2021).

### Intervention characteristics

Training interventions lasted 9 weeks on average (range: 4–16 weeks). The majority of studies used training durations of 4 to 12 weeks, except Ayari et al., (2023) who conducted 16 weeks of interventions. On average, training sessions were performed 2.5 times per week (range: 1–5 sessions per week) and lasted 50 min (range: 30–90 min). Training duration and frequency were matched between the experimental and control groups in all studies.

Intervention adherence rates varied across studies, with only six studies reporting these rates. One study reported a 100% adherence rate for both groups (Tollár et al., 2019). Three studies reported adherence rates between 80 and 90%: Ayari et al. (2023) reported rates of 86.7 and 86.4%, Bisbe et al. (2020) reported 95.1 and 94.9%, and de Rooij et al. (2021) reported 89.3 and 91.7% for the combined training and control groups, respectively. One study (Ferraz et al., 2018) noted similar adherence rates between groups but provided no further details. Swinnen et al. (2024) reported low adherence, with neither group reaching 70%. Only three of the aforementioned studies reported how adherence was measured: Ayari et al. (2023) used questionnaires to assess motivation to attend sessions, while Bisbe et al. (2020) and de Rooij et al. (2021) measured adherence by the percentage of participants attending the intervention sessions. The other 10 studies did not report adherence rates (Baek et al., 2024; Song and Park, 2015; Cano-Mañas et al., 2020; Galperin et al., 2023; Khushnood et al., 2021; Carvalho et al., 2020; Yoon et al., 2013; Lin et al., 2020).

The most frequently used type of combined PT and CT intervention among the included studies was simultaneous-incorporated training by use of video games in nine studies, including Xbox Kinect in six studies (Song and Park, 2015; Cano-Mañas et al., 2020; Ozdogar et al., 2020, 2022; Ferraz et al., 2018; Tollár et al., 2019), and Nintendo Wii in two studies (Khushnood et al., 2021; Carvalho et al., 2020). Additionally, the VITAAL exergaming system was used in one study (Swinnen et al., 2024). These interventions encompassed a variety of interactive video games, involving tasks such as responding to visual stimuli, target shooting, and full-body movement to enhance spatial orientation and visual and working memory. Furthermore, three studies incorporated PT with a virtual reality (VR) component. Among these, two studies involved walking with different interactive virtual components (de Rooij et al., 2021; Galperin et al., 2023), wherein participants engaged in treadmill walking within a 3D environment with obstacles and distractors, necessitating adjustments in steps, attentional control, and response selection. One study focused on rehabilitation exercises with VR, where in the immersive environment, participants performed upper limb strengthening exercises, coordination and cognitive tasks—performing basic mathematical calculations, serial number arrangement according to value, and color discrimination (Lin et al., 2020). Two studies also utilized dancing as a naturally combined form of PT and CT training (Bisbe et al., 2020; Ayari et al., 2023). Finally, two studies used simultaneous (independent) training and sequential training; Baek et al. (2024) and Yoon et al. (2013), respectively. In the simultaneous (independent) training, participants performed resistance training on a machine and performed dual-tasks such as writing names, drawing pictures, and subtracting numbers while performing (Baek et al., 2024). While in the sequential training, participants engaged in stationary cycling along with memory training in a separate, consecutive session (Yoon et al., 2013).

The control group interventions mostly consisted of PT alone (*n* = 15) (Baek et al., 2024; Bisbe et al., 2020; Song and Park, 2015; Ayari et al., 2023; Cano-Mañas et al., 2020; de Rooij et al., 2021; Galperin et al., 2023; Khushnood et al., 2021; Ozdogar et al., 2020, 2022; Carvalho et al., 2020; Ferraz et al., 2018; Swinnen et al., 2024; Tollár et al., 2019; Lin et al., 2020). This included strength training in two studies (Baek et al., 2024; Ayari et al., 2023), stationary cycling in two studies (Song and Park, 2015; Tollár et al., 2019), and treadmill walking in three studies (de Rooij et al., 2021; Galperin et al., 2023; Swinnen et al., 2024). Additionally, conventional rehabilitation exercises and physical therapy were utilized in six studies (Bisbe et al., 2020; Cano-Mañas et al., 2020; Ozdogar et al., 2020, 2022; Ferraz et al., 2018; Lin et al., 2020), while two studies incorporated various forms of stretching and coordination/flexibility exercises (Khushnood et al., 2021; Carvalho et al., 2020). Furthermore, three studies each employed two control groups: one stationary cycling group and the other functional training (Ferraz et al., 2018), another had stationary cycling and a waitlist group (Tollár et al., 2019) and the other had conventional rehabilitation and a waitlist group (Ozdogar et al., 2020). Finally, only one study used CT as a control group, focusing on memory training (Yoon et al., 2013).

Delivery mode of the combined PT and CT interventions was individualized in 11 studies and adapted to the individual level (Song and Park, 2015; Cano-Mañas et al., 2020; de Rooij et al., 2021; Galperin et al., 2023; Khushnood et al., 2021; Ozdogar et al., 2020, 2022; Carvalho et al., 2020; Ferraz et al., 2018; Swinnen et al., 2024; Lin et al., 2020), while it was delivered within a group in four studies (Baek et al., 2024; Bisbe et al., 2020; Ayari et al., 2023; Tollár et al., 2019). One study (Yoon et al., 2013) did not report how the combined PT and CT was delivered. Regarding the control group, eight studies delivered training individually (Song and Park, 2015; Cano-Mañas et al., 2020; de Rooij et al., 2021; Galperin et al., 2023; Khushnood et al., 2021; Ferraz et al., 2018; Swinnen et al., 2024; Ozdogar et al., 2022). Conversely, five studies delivered training in groups (Baek et al., 2024; Bisbe et al., 2020; Ayari et al., 2023; Carvalho et al., 2020; Tollár et al., 2019). Three studies did not specify the delivery mode for the control group (Galperin et al., 2023; Ozdogar et al., 2020, 2022).

Among the studies that reported exercise intensity (*n* = 10), intensity was generally comparable between groups. It ranged from low to moderate, with some interventions showing no progression and others demonstrating progressively increased intensity. Intensity was assessed using various measures: maximum heart rate in three studies (Ayari et al., 2023; Ferraz et al., 2018; Tollár et al., 2019), one-repetition maximum (1RM) in one study (Baek et al., 2024), and rate of perceived exertion (RPE) in another study (Bisbe et al., 2020). In five studies (de Rooij et al., 2021; Galperin et al., 2023; Ozdogar et al., 2020, 2022; Lin et al., 2020), intensity was manipulated through parameters such as speed, dual-task load, repetitions, and number of sets. Six studies did not explicitly report any measure of exercise intensity (Song and Park, 2015; Cano-Mañas et al., 2020; Khushnood et al., 2021; Carvalho et al., 2020; Yoon et al., 2013; Swinnen et al., 2024).

### Depression symptoms outcome measure

The measurement of depression symptoms varied across the included studies. Thirteen studies utilized dedicated depression scales (Baek et al., 2024; Bisbe et al., 2020; Song and Park, 2015; Ayari et al., 2023; de Rooij et al., 2021; Galperin et al., 2023; Ozdogar et al., 2020, 2022; Yoon et al., 2013; Ferraz et al., 2018; Swinnen et al., 2024; Tollár et al., 2019; Lin et al., 2020) whereas three studies used related but not dedicated depression scales (Cano-Mañas et al., 2020; Khushnood et al., 2021; Carvalho et al., 2020). Out of the 13 studies, four studies utilized the Geriatric Depression Scale (GDS) (Baek et al., 2024; Ayari et al., 2023; Yoon et al., 2013; Ferraz et al., 2018), with one study (Ferraz et al., 2018) employing the 15-item version (GDS-15) and the others using the 30-item version (GDS-30). Another four studies utilized the Hospital Anxiety and Depression Scale (HADS) (Bisbe et al., 2020; de Rooij et al., 2021; Ozdogar et al., 2022; Lin et al., 2020). The Beck Depression Inventory (BDI) was used in three studies (Song and Park, 2015; Ozdogar et al., 2020; Tollár et al., 2019). Two studies (Galperin et al., 2023; Swinnen et al., 2024) used the Patient Health Questionnaire (PHQ) and the Cornell scale for depression in dementia (CSDD), respectively. Of the three studies that used scales that are not dedicated depression scales, two studies (Cano-Mañas et al., 2020; Khushnood et al., 2021) utilized the European Quality of Life-5 Dimensions (EQ-5D) and one study (Carvalho et al., 2020) utilized the Fibromyalgia Impact Questionnaire (FIQ). A higher score represents more severe depression symptoms in all the depression rating scales.

### Intervention effects on depression symptoms

Table 2 presents the mean and standard deviation of depression symptom scores at baseline and post-intervention, as well as mean differences. Across the 16 included studies, findings were heterogeneous. Six studies (37.5%) reported significant within-group reductions in depression symptoms following both combined PT and CT and single PT interventions (Baek et al., 2024; Song and Park, 2015; Ayari et al., 2023; Cano-Mañas et al., 2020; Khushnood et al., 2021; Tollár et al., 2019). Five studies (31.2%) reported no significant improvements in depression symptoms in either intervention group (Bisbe et al., 2020; de Rooij et al., 2021; Ferraz et al., 2018; Swinnen et al., 2024; Ozdogar et al., 2022). The remaining studies (31.2%) showed improvements in depression symptoms following combined PT and CT interventions only, but not after single-modality interventions (76, 77,79, 85 for PT; 80 for CT). No significant reductions were reported in passive control groups (Ozdogar et al., 2020; Tollár et al., 2019).

### Decrease in depression symptoms with combined versus single trainings—interactions and standardized mean differences

Only two studies (simultaneously incorporated training) reported a significant time by group interaction favoring the combined intervention over single PT (Carvalho et al., 2020; Lin et al., 2020). Two studies did not find a significant interaction effect (Baek et al., 2024; Ozdogar et al., 2022), see also Table 2. The overall pooled effect size (combined versus single) was SMD = −0.40, 95% CI [−0.69, −0.11], *I*^2^ = 69.7%. Figure 2 shows the individual SMDs for each study. SMD of six studies demonstrated a more pronounced impact of the combined PT and CT intervention over single PT (Baek et al., 2024; Song and Park, 2015; Ayari et al., 2023; Cano-Mañas et al., 2020; Khushnood et al., 2021; Carvalho et al., 2020) and over single CT (Yoon et al., 2013). Additionally, four studies showed a showed a statisitcally significant effect (Table 2) favoring the combined PT and CT intervention over single PT, all *p* <0.05 (Galperin et al., 2023; Ozdogar et al., 2020; Lin et al., 2020; Carvalho et al., 2020). Four studies favored single PT over the combined PT and CT with a non-significant small effect size (Bisbe et al., 2020; de Rooij et al., 2021; Ozdogar et al., 2020; Ferraz et al., 2018). Finally, one study showed no group differences at all (Ozdogar et al., 2022).

**Figure 2:**
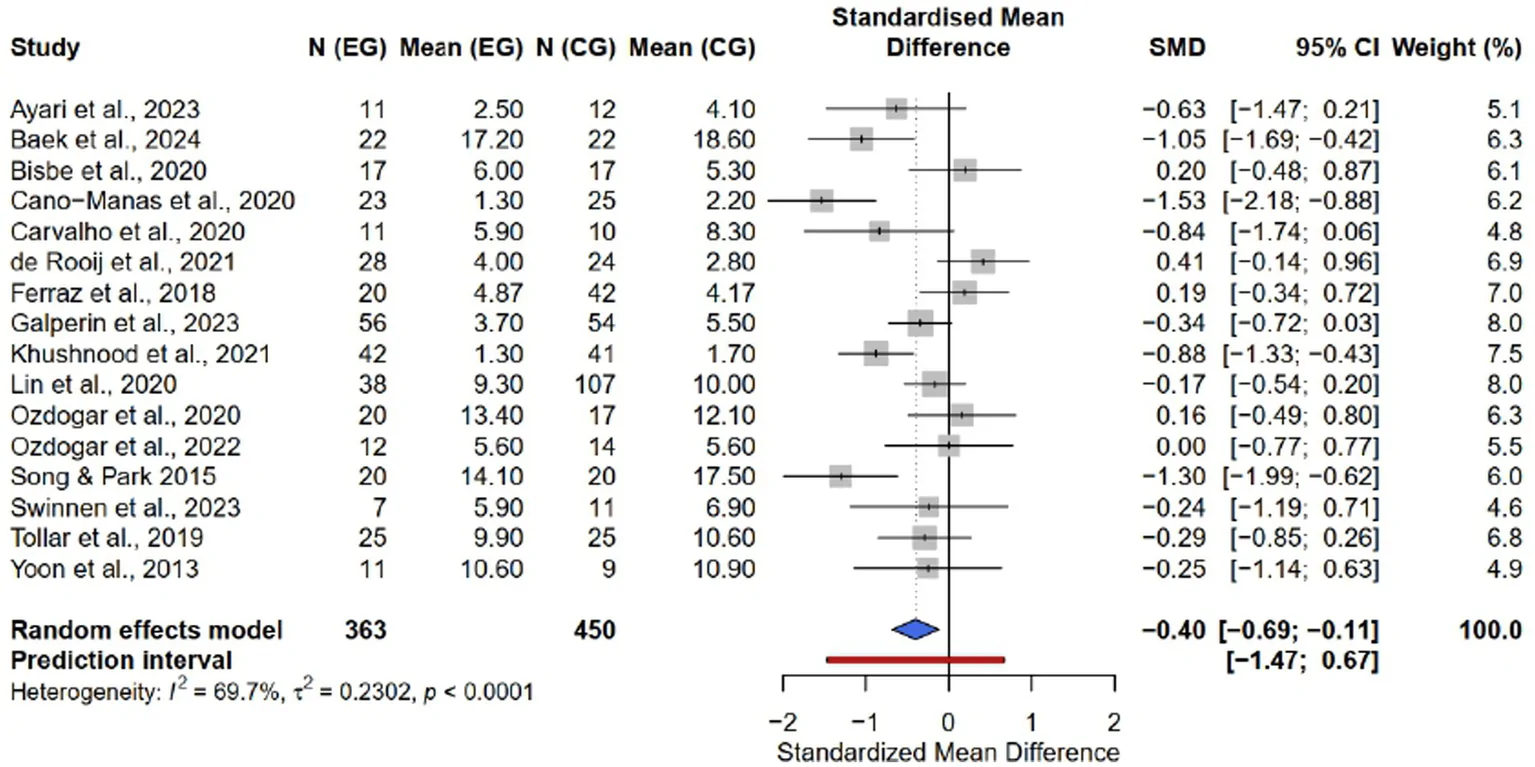
Forest plot of the effect sizes for combined PT and CT versus either single PT or CT on depressive symptoms. An effect size below zero favours the combined PT and CT interventions over single PT or single CT. EG, Experimental Group; CG: Control Group; SMD: Standardized Mean Difference; CI: Confidence Interval.

### Heterogeneity

Moderate heterogeneity was observed across the included studies (*I*^2^ = 69.7%, *p* < 0.001), indicating variability in effect sizes beyond what would be expected by chance.

### Subgroup analysis

The subgroup analysis by intervention type (cf. Figure 3; Table 3) revealed substantial within-subgroup heterogeneity. Studies employing simultaneous incorporated training (exergames) showed high heterogeneity (*I*^2^ = 71%, *p* < 0.001). Despite this substantial within-subgroup heterogeneity, the random-effects test for subgroup differences was not statistically significant (Q_between_ = 3.98, df = 2, *p* = 0.14), suggesting that intervention type did not explain a meaningful proportion of between-study variance. With respect to clinical characteristics (cf. Figure 4; Table 3), studies involving participants with Parkinson’s disease and mild cognitive impairment (MCI) showed small- to- moderate heterogeneity (*I*^2^ = 33.8%, *I*^2^ = 59.5%, respectively all *p* > 0.05). Conversely, studies involving stroke populations exhibited substantial heterogeneity (*I*^2^ = 89.3%, *p* < 0.001), whereas studies including individuals with multiple sclerosis showed no evidence of heterogeneity (*I*^2^ = 0%, *p* > 0.05). Similar to intervention type, the random-effects test for subgroup differences based on clinical characteristics was not statistically significant (Q_between_ = 9.56, df = 6, *p* = 0.14), suggesting that clinical diagnosis did not significantly moderate the pooled effect. Finally, subgroup analyses based on depression assessment scales revealed differing levels of heterogeneity (cf. Figure 5; Table 3). Low heterogeneity was found for studies using HADS (*I*^2^ = 4.6%, *p* = 0.37). Moderate heterogeneity was observed among studies using the GDS (*I*^2^ = 67.2%, *p* = 0.03). Substantial heterogeneity was evident for studies using the BDI (*I*^2^ = 79.1%, *p* < 0.01). Moderate heterogeneity was observed for studies using the EQ-5D (*I*^2^ = 61.8%, *p* = 0.11). Importantly, the random-effects test for subgroup differences was statistically significant (Q_between_ = 14.08, df = 6, *p* = 0.03), indicating that effect sizes differed significantly across depression scales.

**Figure 3:**
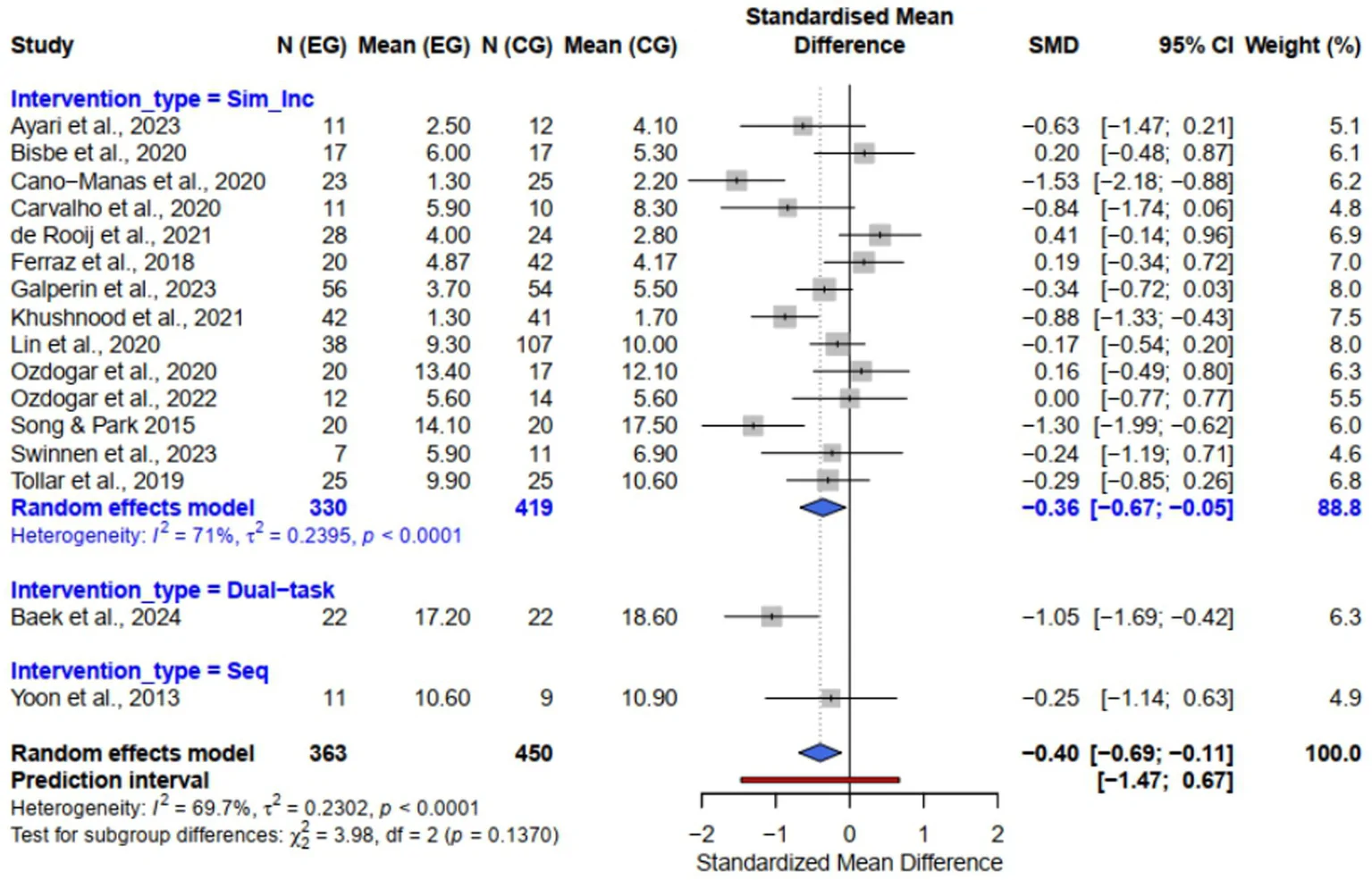
Forest plot of the effect sizes for combined PT and CT versus either single PT or CT on depressive symptoms separated by intervention type. An effect size below zero favours the combined PT and CT interventions over single PT or single CT. EG: Experimental Group; CG: Control Group; SMD: Standardized Mean Difference; CI: Confidence Interval; Simultaneous incorporated training; Seq: Sequential training; Dual-task (also refers to simultaneous independent training).

**Table 3:** Subgroup and sensitivity analysis to explore the potential sources of heterogeneity and its impact on the robustness of the findings.

Analysis type	Category	Included studies	Heterogeneity (*I*^2^) and Robustness of Findings
Subgroup analysis (delivery mode)	Simultaneous- incorporated training (exergames)	Ayari et al. (2023), Bisbe et al. (2020), Cano-Mañas et al. (2020), Carvalho et al. (2020), de Rooij et al. (2021), Ferraz et al. (2018), Galperin et al. (2023), Khushnood et al. (2021), Lin et al. (2020), Ozdogar et al. (2020, 2022), Song and Park (2015), Swinnen et al. (2024), Tollár et al. (2019)	*I*^2^ = 68.6%; *p* < 0.001
	Simultaneous independent training (dual-tasking)	Baek et al. (2024)	
	Sequential training	Yoon et al. (2013)	
Subgroup analysis (clinical characteristics)	MCI	Ayari et al. (2023), Swinnen et al. (2024), Baek et al. (2024), Bisbe et al. (2020)	*I*^2^ = 59.5%; *p* = 0.06
	Dementia	Yoon et al. (2013)	
	Stroke	Cano-Mañas et al. (2020), de Rooij et al. (2021), Lin et al. (2020), Song and Park (2015)	*I*^2^ = 89.3%; *p* < 0.001
	Parkinson’s Disease	Ferraz et al. (2018), Tollár et al. (2019), Cano-Mañas et al. (2020)	*I*^2^ = 0%; *p* = 0.71
	Multiple Sclerosis	Galperin et al. (2023), Ozdogar et al. (2020, 2022)	*I*^2^ = 0%; *p* = 0.38
	Fibromyalgia	Carvalho et al. (2020)	
	Healthy	Khushnood et al. (2021)	
Subgroup analysis (depression scale)	GDS	Ayari et al. (2023), Baek et al. (2024), Baek et al. (2024), Ferraz et al. (2018), Yoon et al. (2013)	*I*^2^ = 40.2%; *p* = 0.17
	HADS	Bisbe et al. (2020), de Rooij et al. (2021), Lin et al. (2020), Ozdogar et al. (2022)	*I*^2^ = 4.6%; *p* = 0.37
	EQ-5D	Cano-Mañas et al. (2020), Khushnood et al. (2021)	*I*^2^ = 61.8%; *p* = 0.11
	FIQ	Carvalho et al. (2020)	
	PHQ	Galperin et al. (2023)	
	BDI	Ozdogar et al. (2020), Song and Park (2015), Tollár et al. (2019)	*I*^2^ = 79.1%; *p* = 0.01
	CSDD	Swinnen et al. (2024)	
Sensitivity analysis	13 included studies (restricted to dedicated depressive scales)		*I*^2^ = 57.2; *p* < 0.01 Overall SMD = −0.24 (95% CI, −0.51 to 0.03).

SMD denotes standardized mean difference. Robustness inferred when statistical significance is retained after sensitivity analysis. GDS, Geriatric Depression Scale; HADS, Hospital Anxiety and Depression Scale; EQ-5D, European Quality of Life-5 Dimensions; FIQ, Fibromyalgia Impact Questionnaire; PHQ, Patient Health Questionnaire; BDI, Beck Depression Inventory; CSDD, Cornell Scale for Depression in Dementia.

**Figure 4:**
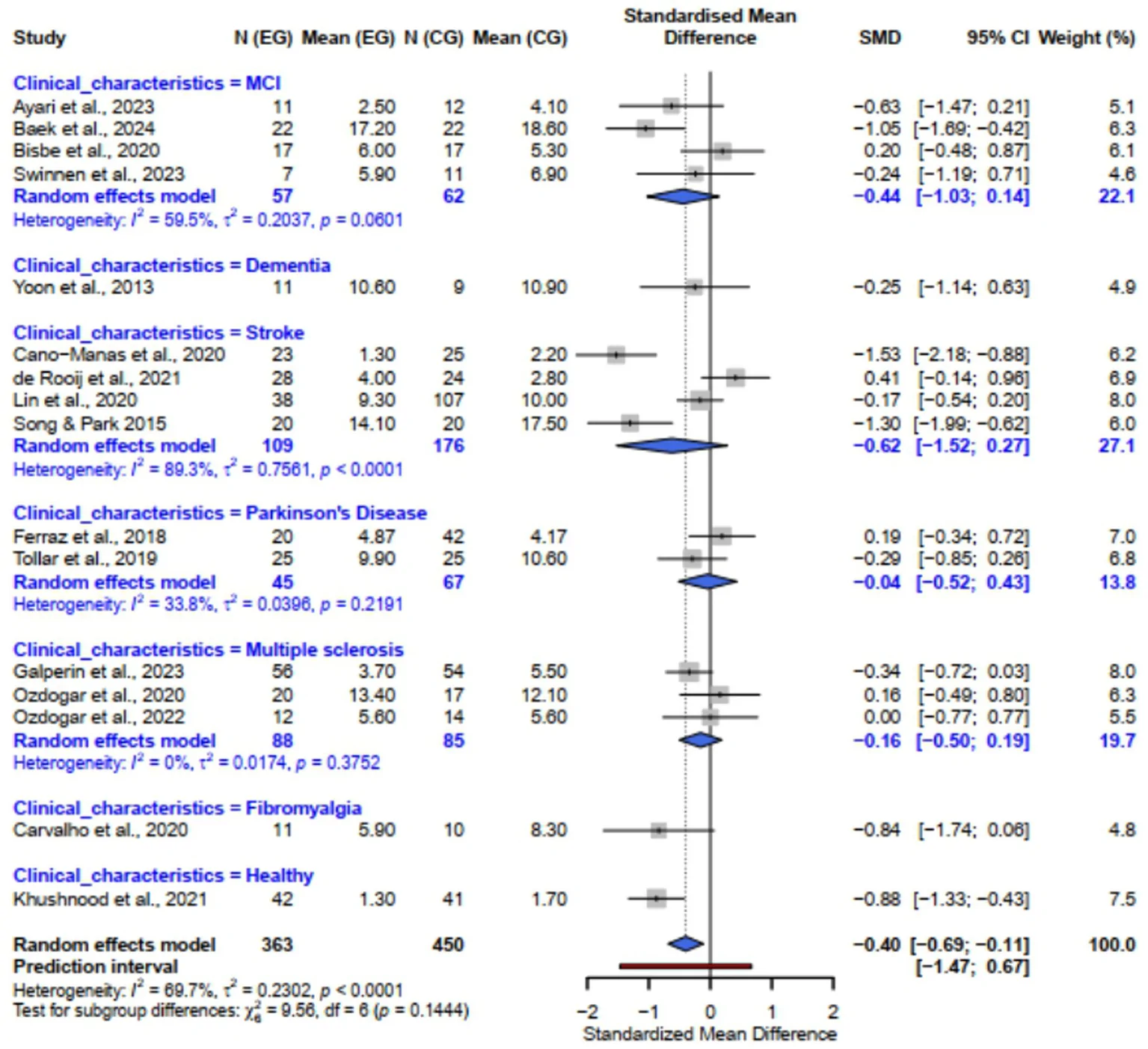
Forest plot of the effect sizes for combined PT and CT versus either single PT or CT on depressive symptoms separated by participants’ clinical characteristics. Note: An effect size below zero favours the combined PT and CT interventions over single PT or single CT. EG, Experimental group; CG, Control group; SMD, Standardized mean difference; CI, Confidence interval; MCI, Mild cognitive impairment.

**Figure 5:**
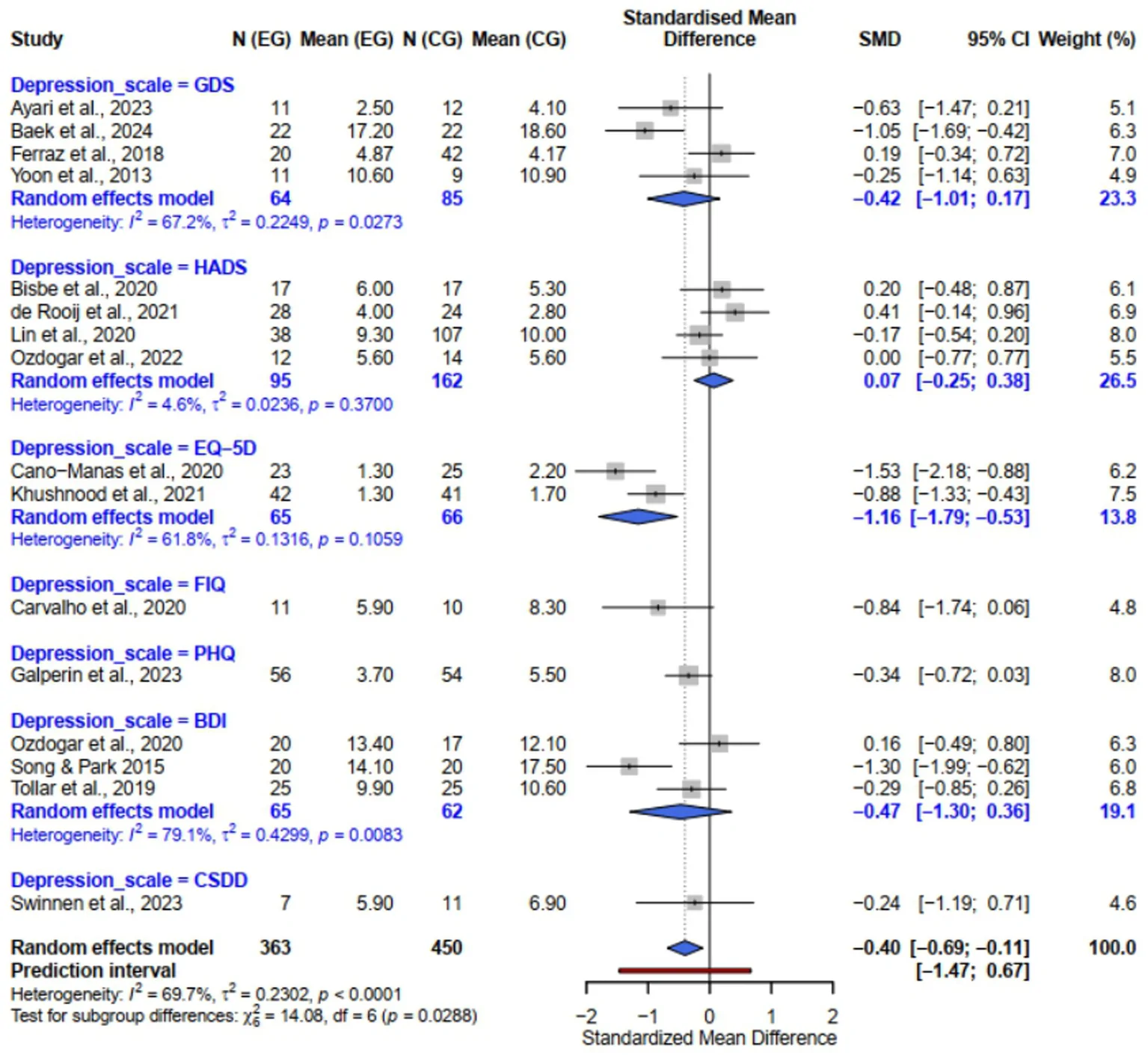
Forest plot of the effect sizes for combined PT and CT versus either single PT or CT on depressive symptoms separated by depression scale. An effect size below zero favours the combined PT and CT interventions over single PT or single CT. EG, Experimental group; CG, Control group; SMD, Standardized mean difference; CI, Confidence interval; GDS, Geriatric depression scale; HADS, Hospital anxiety and depression scale; EQ-5D, European quality of life-5 dimensions; FIQ, Fibromyalgia impact questionnaire; PHQ, Patient health questionnaire; BDI, Beck depression inventory; CSDD, Cornell scale for depression in dementia.

### Sensitivity analysis

A sensitivity analysis restricted to the 13 studies (cf. Figure 6) that used dedicated depression symptom measures (Baek et al., 2024; Bisbe et al., 2020; Song and Park, 2015; Ayari et al., 2023; de Rooij et al., 2021; Galperin et al., 2023; Ozdogar et al., 2020, 2022; Yoon et al., 2013; Ferraz et al., 2018; Swinnen et al., 2024; Tollár et al., 2019; Lin et al., 2020) yielded a smaller pooled effect favoring combined PT and CT interventions over single-modality interventions; however, the effect was no longer statistically significant (SMD = −0.24, 95% CI [−0.51, 0.03], *I*^2^ = 57.2%). Although the direction of the pooled effect remained unchanged, these findings indicate that the estimated treatment effect was attenuated when analyses were restricted to depression-specific outcome measures, highlighting the influence of outcome measurement on the pooled estimates. Similarly, a sensitivity analysis restricted to the six studies with an overall low risk of bias (Bisbe et al., 2020; de Rooij et al., 2021; Galperin et al., 2023; Ferraz et al., 2018; Tollár et al., 2019; Lin et al., 2020) yielded no statistically significant difference between combined PT and CT and single-modality interventions (SMD = −0.05, 95% CI − 0.30 to 0.19; cf. Supplementary materials, Figure 1).

**Figure 6:**
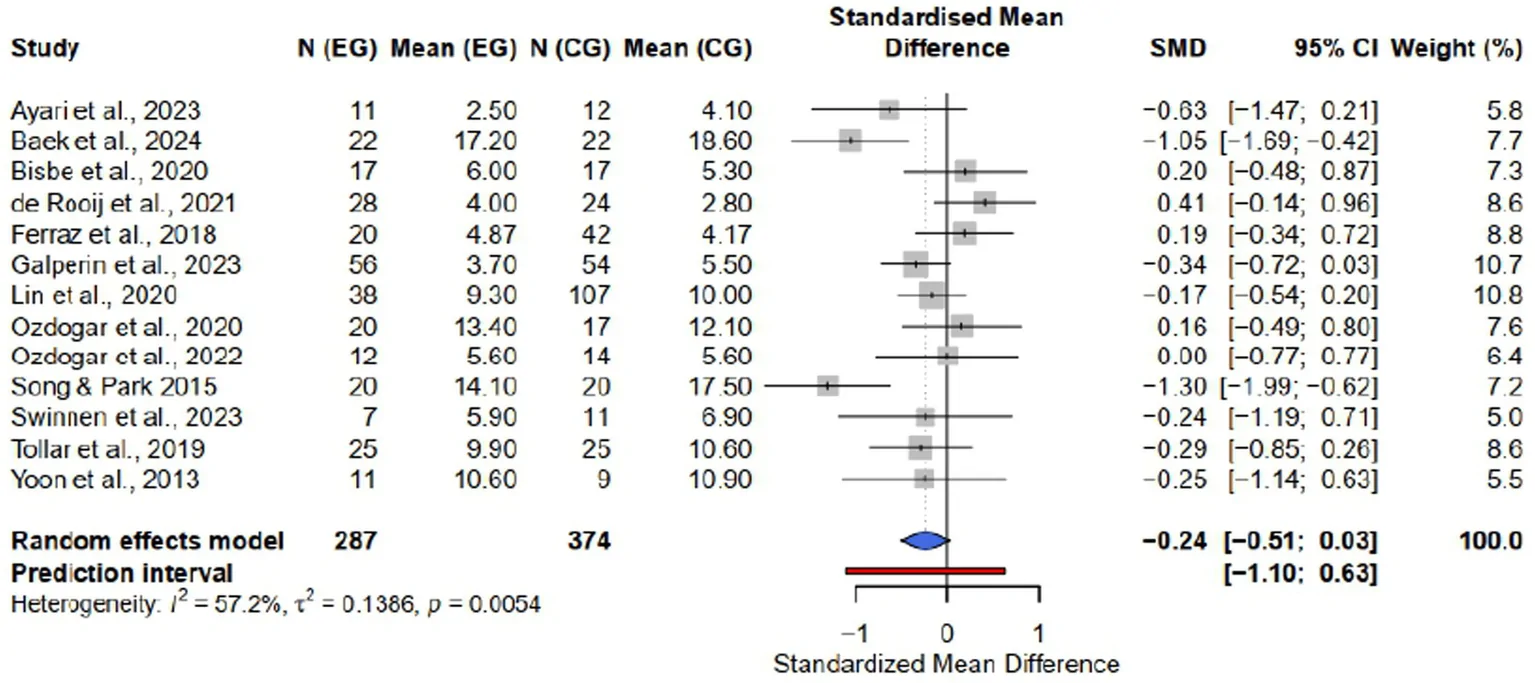
Forest plot of the effect sizes restricted to dedicated depressive scales only (sensitivity analyses). An effect size below zero favours the combined PT and CT interventions over PT alone. EG, Experimental group; CG, Control group; SMD, Standardized mean difference; CI, Confidence interval.

### Publication bias

Egger’s regression test (cf. Figure 7) showed an intercept estimate of −0.31 (95% CI − 1.19 to 0.57), indicating no evidence of funnel plot asymmetry (z = −0.31, *p* = 0.75).

**Figure 7:**
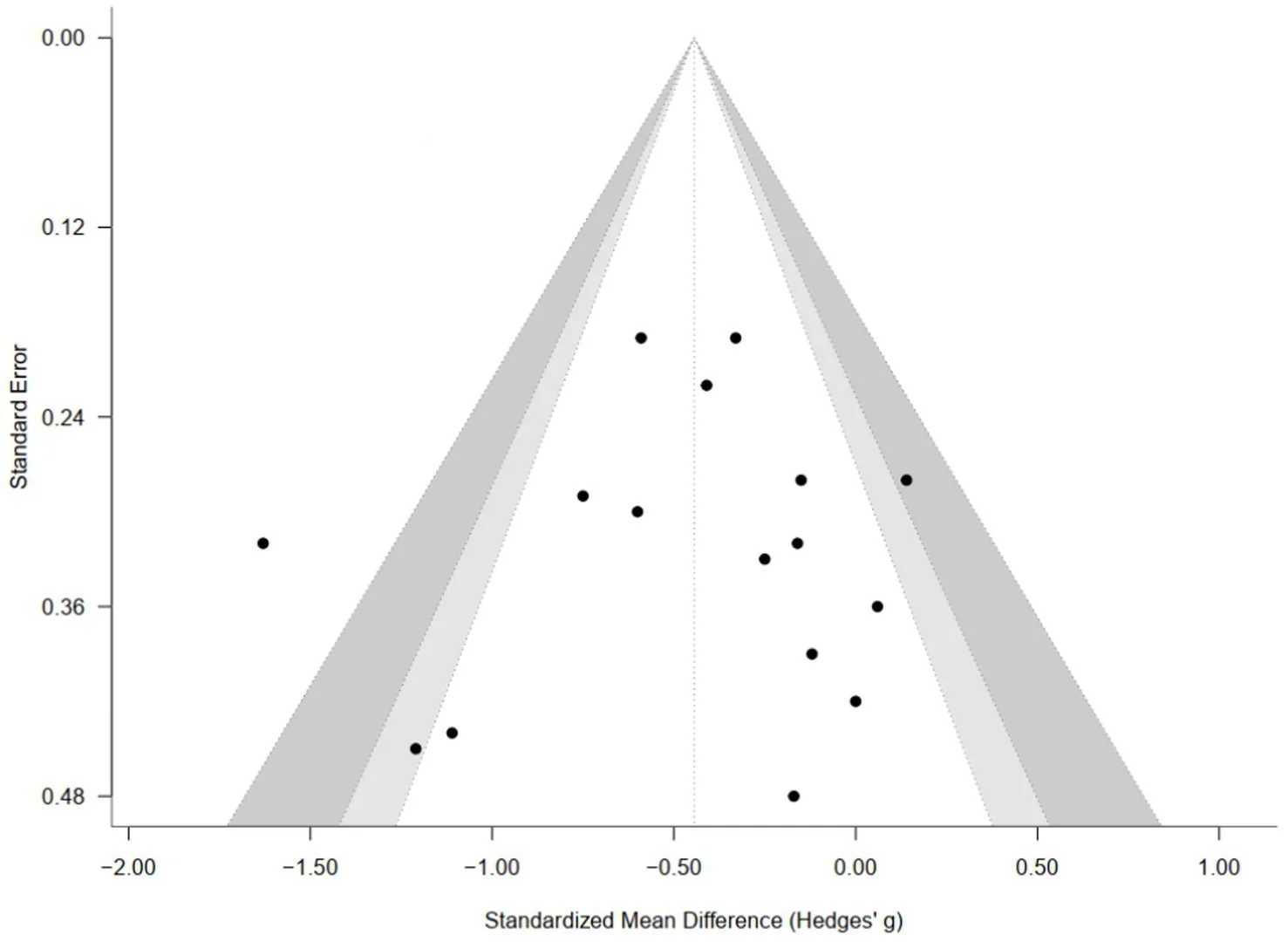
Funnel plot of studies comparing combined PT and CT interventions with either single PT or single CT interventions. Funnel plot used to assess potential small-study effects on the pooled effect size. Each dot represents an individual study.

### Risk of bias in studies

In our risk of bias assessment, 10 of the 16 included studies were judged to have some concerns overall (Baek et al., 2024; Song and Park, 2015; Ayari et al., 2023; Cano-Mañas et al., 2020; Khushnood et al., 2021; Ozdogar et al., 2020; Carvalho et al., 2020; Yoon et al., 2013; Swinnen et al., 2024; Ozdogar et al., 2022), while six studies were assessed as having a low overall risk of bias (Bisbe et al., 2020; de Rooij et al., 2021; Galperin et al., 2023; Ferraz et al., 2018; Tollár et al., 2019; Lin et al., 2020). Concerns primarily related to the randomization process (D1) and measurement of the outcome (D4). Additional concerns included missing outcome data (D3) in Carvalho et al. (2020) and Swinnen et al. (2024), deviations from the intended intervention (D2) in Swinnen et al. (2024), and selection of the reported result (D5) in Song and Park (2015). Figure 8 presents detailed domain-level risk-of-bias judgments for each study.

**Figure 8:**
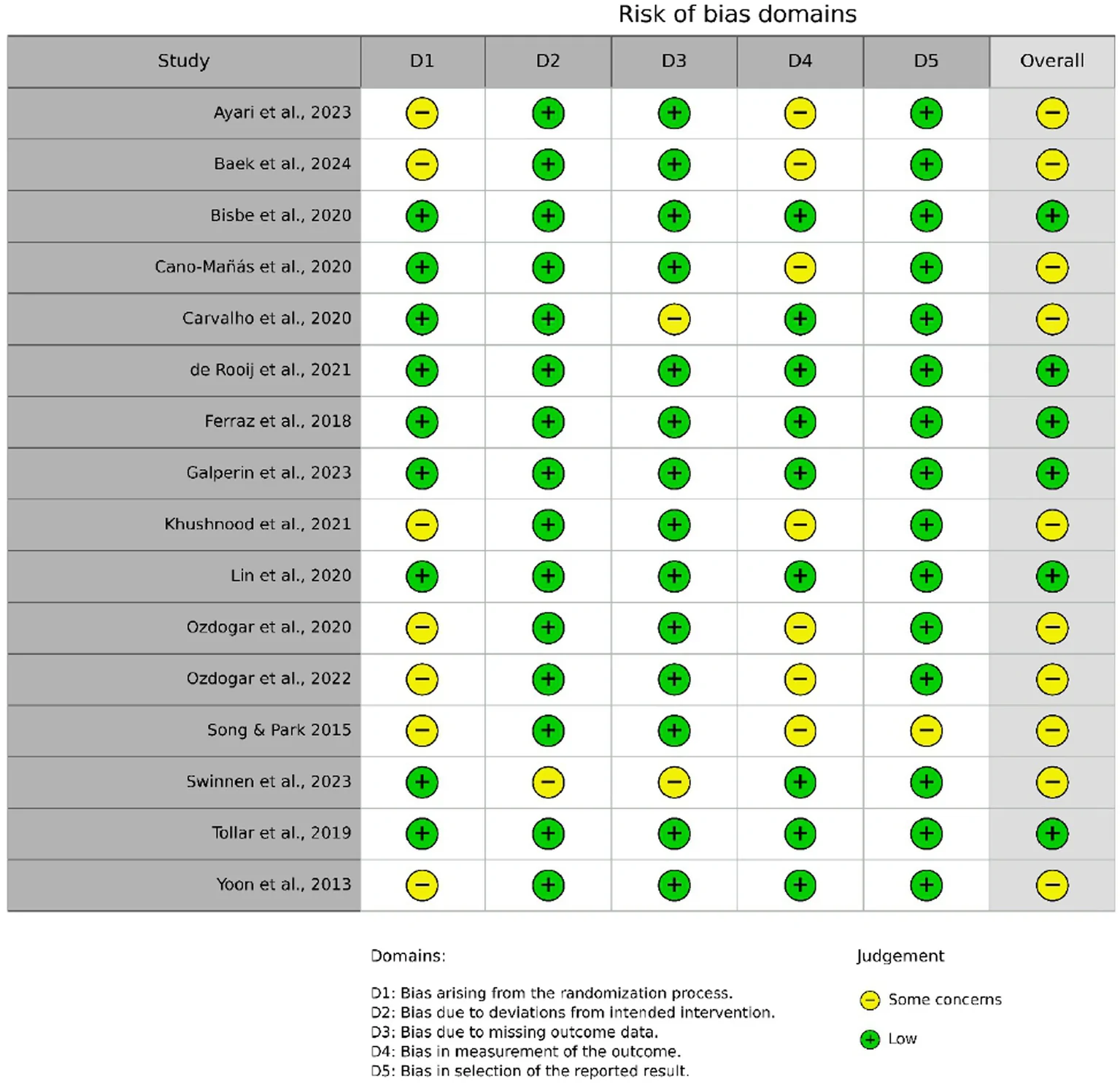
Risk of bias assessment showing the internal validity of the included studies.

### Certainty of evidence (GRADE)

The certainty of evidence for depression symptoms was rated as very low according to the GRADE approach. The evidence was downgraded due to inconsistency (moderate heterogeneity) and indirectness (predominantly heterogeneous clinical populations with neurological and medical comorbidities rather than primary depressive disorder samples) (cf. Supplementary materials, Table 4).

## Discussion

### Summary and interpretation

This systematic review provides the first comprehensive synthesis of RCTs comparing combined PT and CT with single-modality interventions for reducing depression symptoms in adults. Across all 16 RCTs, pooled between-group analyses indicated that combined PT and CT interventions, including simultaneous-incorporated training, simultaneous (independent) training, and sequential training, were associated with greater reductions in depression symptoms than PT or CT alone (SMD = −0.40, 95% CI [−0.69, −0.11], *I*^2^ = 69.7%). However, when the analysis was restricted to studies using depression-specific outcome measures, the pooled effect was attenuated and no longer statistically significant (SMD = −0.24, 95% CI [−0.51, 0.03], *I*^2^ = 57.2%), suggesting that outcome measurement may have influenced the estimated effect. At the individual study level, both combined and single-modality interventions were frequently associated with reductions in depression symptoms; however, the magnitude of improvement varied across studies. Importantly, the available evidence was derived predominantly from comparisons with PT alone (15 of the 16 included studies), with only one study directly comparing combined interventions with CT alone.

Given the moderate heterogeneity observed in both primary and sensitivity analyses, subgroup analyses by intervention type, participants’ clinical characteristics and depression scales were performed. Neither intervention type nor clinical characteristics significantly explained between-study variability, despite substantial within-study heterogeneity. Conversely, depression scales (outcome measurement) emerged as a meaningful source of heterogeneity: studies using the GDS and HADS showed low and consistent variability, whereas those using the BDI and EQ-5D exhibited moderate to substantial heterogeneity, suggesting that differences in depression assessment tools contribute to variability in the observed effect estimates. This interpretation is supported by the sensitivity analysis, in which restricting the analysis to studies using depression-specific instruments resulted in a smaller pooled effect that was no longer statistically significant.

Nevertheless, the observed heterogeneity is likely to be multifactorial. Beyond differences in outcome measurement, the included studies varied considerably in intervention protocols, including training frequency, duration, cognitive task complexity, exercise content, delivery format, participant characteristics, and comparator interventions. While subgroup analyses explored several potential sources of heterogeneity individually, they could not account for the combined influence or interaction of these study characteristics. Consequently, the moderate heterogeneity observed in this review likely reflects the cumulative effect of multiple methodological and clinical differences across studies rather than a single dominant source.

Importantly, no evidence of funnel-plot asymmetry was detected, suggesting that the overall pooled effect estimates are unlikely to be driven by selective publication of smaller studies with inflated effects, thereby strengthening confidence in the robustness of the overall findings despite moderate heterogeneity.

Taken together, the primary analysis suggests that combined PT and CT interventions may be associated with greater reductions in depression symptoms than single-modality approaches. However, the attenuation and loss of statistical significance observed in the sensitivity analysis indicate that this finding is sensitive to the type of depression outcome measure used. Consequently, although the direction of the pooled effect remained consistent, confidence in the magnitude and robustness of the pooled effect is reduced.

Our systematic review is the first to exclusively compare the effects of combined PT and CT interventions on depression symptoms versus PT or CT alone, preventing direct comparisons with prior studies on this specific outcome. However, our findings align with previous evidence showing the greater effect of a combined approach over single training methods for cognitive performance (Dhir et al., 2021; Gheysen et al., 2018; Rieker et al., 2022). Although these studies used different outcome measures and samples, their findings may relate to depression symptoms, as discussed in the introduction. Additionally, our results build upon Huang et al. (2022), who found that combined training in the form of exergaming reduced depression symptoms in adults more than active and passive controls, even though the prediction interval did not reach statistical significance. Our review extends these findings by suggesting that various combined approaches, including simultaneous (independent), simultaneous-incorporated, and sequential PT and CT interventions, may be associated with greater reductions in depression symptoms than PT or CT alone. However, this apparent advantage should be interpreted cautiously because the pooled effect was attenuated and no longer statistically significant when analyses were restricted to studies using depression-specific outcome measures.

### Potential factors affecting the impact of combined PT and CT interventions: dose–response, intensity of cognitive training, and disease type

We attempted to identify patterns in the available studies that may explain the observed effects of combined PT and CT on depression symptoms compared to single PT or CT. We focused on training parameters (e.g., type, frequency, duration), the specific cognitive and physical components, and sample characteristics (e.g., disease type). However, the interventions varied considerably across studies, making it difficult to establish a consistent pattern. Therefore, the following observations should be considered exploratory and interpreted with caution.

First, there is some indication that dose–response might play a role. For example, Cano-Mañas et al. (2020) and Song and Park (2015) reported reductions in depression symptoms associated with combined PT and CT intervention compared to PT alone or CT alone. However, Ozdogar et al. (2020, 2022) used the same exergame system (Kinect Xbox), yet found no difference. One possible explanation for the inconsistent findings is variation in training frequency and duration. Whiles Cano-Mañas et al. (2020) and Song and Park (2015) implemented more frequent and prolonged interventions: 90 min three times per week for 8 weeks or 30 min five times per week for 8 weeks, respectively, Ozdogar et al. (2020, 2022) studies involved 45 min once per week for 8 weeks and 45 min once per day during hospitalization, respectively. Similarly, Lin et al. (2020) reported benefits with frequent and prolonged training (75 min five times per week for 8 weeks), while de Rooij et al. (2021), with shorter and less frequent sessions (30 min twice per week for 6 weeks), did not. Although these observations are consistent with the possibility that intervention dosage may influence treatment effects, they are based on indirect comparisons between studies and should not be interpreted as evidence of a dose–response relationship. Formal dose–response meta-analyses or meta-regression are required to evaluate this hypothesis.

Second, the observed effects may depend on the intensity of the cognitive and physical components (Stojan and Voelcker-Rehage, 2019). In studies showing benefits of combined PT and CT interventions, participants engaged in demanding and varying cognitive tasks such as executive function, math calculations, color discrimination, and memory training (Song and Park, 2015; Cano-Mañas et al., 2020; Carvalho et al., 2020; Yoon et al., 2013; Lin et al., 2020). By contrast, studies that did not find advantages of combined PT and CT (Bisbe et al., 2020; Ayari et al., 2023) included fewer demanding tasks with a low-to-moderate PT component, such as following dance steps. The intensity of the PT was low to moderate in all these studies and thus might not have been the driving factor.

Third, we found no consistent pattern of evidence that the potential benefits of combined PT and CT training are dependent on participants’ clinical characteristics. Six studies reporting positive effects included depressed patients with diverse comorbidities (MCI, stroke, fibromyalgia, dementia) (Baek et al., 2024; Song and Park, 2015; Cano-Mañas et al., 2020; Carvalho et al., 2020; Lin et al., 2020). Similarly, a study with healthy participants (Khushnood et al., 2021) also favored combined PT and CT intervention. This contrasts with a review of Huang et al. (2022), who reported robust effects in stroke patients but less consistent findings in other populations, including those without clinical conditions.

Taken together, the current evidence highlights several intervention characteristics, including training frequency, duration, intensity, and task complexity, as plausible factors that may contribute to the effectiveness of combined PT and CT interventions. However, because these observations are based on qualitative comparisons across heterogeneous studies rather than formal moderator analyses, and because the pooled effect was attenuated and no longer statistically significant when analyses were restricted to depression-specific outcome measures, they should be interpreted cautiously and viewed as hypotheses for future research. Future studies employing harmonized intervention protocols and formal dose–response or meta-regression analyses are needed to determine the independent contribution of these intervention characteristics.

### Potential neurobiological mechanisms underlying the synergetic effects of combined PT and CT in reducing depression symptoms

Although the included trials primarily evaluated clinical outcomes rather than neurobiological mechanisms, previous literature provides several plausible explanations for the potentially greater effectiveness of combined PT and CT interventions in reducing depression symptoms. These mechanisms should therefore be regarded as hypothetical and interpreted with caution.

One proposed mechanism is enhanced neuroplasticity through complementary effects of PT and CT (Castells-Sánchez et al., 2019). PT has consistently been associated with increased expression of neurotrophic factors, particularly brain-derived neurotrophic factor (BDNF), which promotes neurogenesis, synaptic plasticity, and long-term potentiation (Castells-Sánchez et al., 2019; Kempermann et al., 2010). Similarly, cognitively demanding activities have been shown to stimulate BDNF expression, strengthen synaptic plasticity, and facilitate the integration and survival of newly generated neurons (Stojan and Voelcker-Rehage, 2019; Gheysen et al., 2018; Nicastri et al., 2021). Consequently, it has been proposed that combining PT and CT may amplify neuroplastic adaptations through complementary biological pathways (Stojan and Voelcker-Rehage, 2019). Among the studies included in this review, only Anderson-Hanley and colleagues reported higher BDNF levels following combined PT and CT compared with PT alone (Anderson-Hanley et al., 2012). However, because most included trials did not assess neurobiological outcomes, the present review cannot determine whether changes in BDNF or neuroplasticity mediated the observed improvements in depression symptoms.

Another plausible mechanism is increased level of cerebral blood flow (CBF). Previous studies have shown that individuals with depression often exhibit reduced CBF (Chiappelli et al., 2023), whereas PT, increases cerebral perfusion and oxygen delivery (Prudente et al., 2023). Preliminary evidence from interventions combining physical and cognitive tasks suggests that such training may enhance cerebral perfusion. For example, Kunieda and Arakawa (Kunieda et al., 2022) reported greater increases in regional CBF, particularly in the bilateral frontal lobes, following 12 months of simultaneous exercise and cognitive dual-task training compared with usual care in older adults with amnestic MCI. These findings provide preliminary support for enhanced cerebral perfusion as a potential neurobiological pathway through which combined PT and CT may influence depression symptoms. However, they do not establish that increased CBF mediates improvements in depression. Indeed, none of the included trials in the present review directly examined CBF as a mediator of changes in depressive symptoms. Thus, the contribution of altered cerebral perfusion to the synergistic effects of combined PT and CT remains speculative.

Overall, existing neurobiological evidence provides plausible mechanistic hypotheses linking combined PT and CT to improvements in depression symptoms. However, these mechanisms were largely not evaluated in the included randomized controlled trials. Future studies should integrate clinical outcomes with neurobiological measures, such as BDNF, cerebral blood flow, or other biomarkers of neuroplasticity, to determine whether these proposed mechanisms mediate the antidepressant effects of combined PT and CT interventions.

### Strengths and limitations of the current review

To the best of our knowledge, our systematic review and meta-analysis is the first to comprehensively screen and update the literature regarding the potentially stronger effects of combined PT and CT interventions over single PT or CT in reducing depression symptoms in adults. Our review has strengths and limitations. The review comprehensively includes a diverse range of combined training interventions, including simultaneous-incorporated training, simultaneous (independent) training, and sequential training. This inclusivity is especially valuable as it ensures that individuals who may lack access to specific interventions due to financial or physical constraints can still benefit from alternative PT and CT strategies. This detailed understanding can also inform health practitioners and the health sector for the development of targeted interventions that maximize therapeutic benefits for individuals with depression symptoms or depression. Additionally, we used a standardized method to assess depression symptoms, using the SMD to analyze data across studies that utilized various psychometric scales. This approach goes beyond systematic review and observation synthesis, allowing us to standardize results and to combine depression scores from multiple studies for a more rigorous and reliable analysis. Furthermore, in our review, we included different populations (both clinical and healthy) with depression symptoms, as we hoped to provide applicable relevance to a wider range of individuals with depression symptoms.

However, the findings of this review should be interpreted in light of several limitations of the included evidence. First, the included studies generally involved relatively small sample sizes. Across the 16 included RCTs, the median sample size was 22 participants in the combined PT and CT groups and 25 participants in the comparator groups, which may have reduced statistical power within individual studies and limits the generalizability of the findings. Second, the included populations were heterogeneous and predominantly comprised adults with comorbid conditions, including dementia, stroke, Parkinson’s disease, fibromyalgia, multiple sclerosis, and mild cognitive impairment. Only one study included healthy adults, and none specifically recruited participants with a clinically diagnosed depressive disorder. Consequently, the applicability of the findings to healthy adults and clinically depressed populations remains uncertain. Thirdly, many studies did not report the group × time interaction effect, limiting direct statistical evidence regarding the comparative advantage of combined PT and CT over single-modality interventions. Furthermore, adherence and compliance with the interventions were inconsistently reported across studies, limiting evaluation of how intervention fidelity affects treatment effectiveness. Some studies also had risk-of-bias concerns related to missing outcome data, deviations from intended interventions, outcome measurement, and baseline imbalances (Figure 8). Although no study was judged to have high overall risk of bias, restricting the analysis to studies at low risk of bias attenuated the effect to non-significance (SMD = −0.05, 95% CI − 0.30 to 0.19) (cf. Supplementary materials, Figure 1), indicating limited robustness of the pooled effect. Finally, the distribution of comparator interventions was highly imbalanced, with only one study comparing combined PT and CT with CT alone (*n* = 9), whereas 15 studies compared combined interventions with PT alone (*n* = 441). Consequently, the evidence supporting the advantage of combined interventions over PT alone is substantially stronger than that for CT alone, and conclusions regarding CT-only comparators should therefore be interpreted with caution.

The review process also has several limitations. First, only peer-reviewed studies published in English were included, which may have introduced language and publication bias despite no evidence of small-study effects or publication bias in the present analyses. Second, although using SMDs facilitated the synthesis of studies employing different outcome instruments, the review included both dedicated depression scales and broader validated instruments containing depression-related components. While methodological guidance supports pooling conceptually comparable outcomes measured using different instruments, this approach assumes that the instruments assess the same underlying construct of depression symptom severity. Differences in psychometric properties, including reliability, sensitivity, responsiveness, and the inclusion of both dedicated depression scales and broader instruments containing depression-related components, may have contributed to the observed between-study heterogeneity. However, only three studies used non-specific instruments, making such a subgroup analysis insufficiently powered to provide reliable estimates. We therefore considered a separate meta-analysis by measurement type inappropriate. Moreover, because the included instruments use different scoring systems and instrument-specific minimally important clinical differences (MCIDs), the pooled standardized effect cannot readily be translated into a clinically meaningful threshold. The attenuation of the pooled effect in the sensitivity analysis restricted to depression-specific measures further suggests that outcome measurement influenced the estimated treatment effect. Consequently, although the findings support an overall beneficial effect of combined physical and cognitive training, both the magnitude and clinical interpretation of the pooled effect should be interpreted with appropriate caution. Third, the quantitative subgroup analyses deviate from the pre-registered PROSPERO protocol, which had planned only qualitative exploration of potential effect modifiers. We conducted these analyses because sufficient data became available, but they should be regarded as exploratory rather than confirmatory. Finally, the relatively small number of included studies, together with substantial variability in intervention characteristics, prevented formal investigation of independent moderators through meta-regression and limited our ability to examine dose–response relationships. Future research should employ standardized depression assessment tools, report intervention adherence consistently, include more direct comparisons with CT-only interventions and clinically diagnosed depressive populations, and, where sufficient studies are available, use meta-regression to investigate intervention characteristics and outcome measurement as potential sources of heterogeneity.

### Implications and future research

Our review highlights several gaps in the literature that future research can address to improve the understanding and impact of combined PT and CT interventions on reducing depression symptoms. Based on our findings, we recommend that future RCTs should consider the following points:

#### Larger sample sizes and diverse participant characteristics

Based on our moderate effect size (−0.4), we recommend that future RCTs include at least 100 participants per group to achieve 80% statistical power at an alpha level of 0.05 and detect an effect size of 0.4. Additionally, future research should compare outcomes between comorbid populations and healthy individuals with depression symptoms. This comparison will help determine if combined PT and CT interventions are effective across different groups or only in cases with comorbid conditions such as dementia or stroke.

#### Standardized reporting of adherence and exercise characteristics

Future studies should standardize the measurement and reporting of adherence and exercise characteristics to ensure consistent and reliable data. Consistent adherence metrics and a reporting of the exercise characteristics according to the FITT principles will improve the interpretation of research findings and their applicability (Wollesen et al., 2025). Neglecting to examine and report both aspects and its relation to outcomes can compromise the interpretation of research findings (Beintner et al., 2019).

#### Exploring neurobiological mechanisms of the PT and CT synergistic effect on depression

Several neurophysiological studies have investigated the effects of combined PT and CT interventions on cognitive performance (Bamidis et al., 2015; Dhir et al., 2021; Gheysen et al., 2018). There is still a gap in knowledge about how these interventions specifically influence the neural circuits associated with depression. Future research should focus on elucidating the neuroanatomical underpinnings of the synergistic effects of combined training on depression networks. Understanding these mechanisms will be crucial for advancing the field and keeping pace with the rapid proliferation of efficacy investigations.

## Conclusion

The current systematic review and meta-analysis suggest that both combined PT and CT and single-modality interventions, predominantly PT, are associated with reductions in depression symptoms. The primary pooled between-group analysis suggested that combined PT and CT interventions may be associated with greater reductions in depression symptoms than single-modality interventions, particularly compared with PT alone. However, this effect was attenuated and no longer statistically significant when the analysis was restricted to studies using depression-specific outcome measures. The certainty of this evidence was rated as very low according to the GRADE framework, indicating limited confidence in the estimated effect. The available evidence is derived predominantly from adults with depression symptoms and comorbid chronic diseases, while direct comparisons with CT-only interventions and evidence from healthy populations remain scarce. Together with the moderate between-study heterogeneity arising from variation in intervention protocols, participant characteristics, and outcome measurement, these limitations warrant cautious interpretation of the findings. Although exploratory observations suggest that intervention characteristics such as training frequency, duration, and cognitive task complexity may influence treatment effects, these findings require confirmation in adequately powered studies. Overall, combined PT and CT interventions appear promising for reducing depression symptoms; however, the current evidence is insufficient to draw robust conclusions regarding their superiority over single-modality interventions. Future research must use standardized measurement tools and rigorous adherence to reporting guidelines (Wollesen et al., 2025) to ensure the generalizability and methodological reliability of these findings across diverse populations.

## Data Availability

The authors will make the raw data supporting the conclusions of this manuscript available upon reasonable request. Requests to access these datasets should be directed to claudia.voelcker-rehage@uni-muenster.de.
