# Supplementary material for: Systematic review and meta-analysis of the effects of combined physical and cognitive training on depression symptoms in adults across clinical populations
Source: Front Psychol. 2026 Sep 24;17:1741014. doi: 10.3389/fpsyg.2026.1741014 (PMC13646151; doi:10.3389/fpsyg.2026.1741014)
Supplement: Supplementary file 1 [file Data_Sheet_1.docx]

Table 1. Inclusion and Exclusion Criteria

| PICOS | Inclusion Criteria | Note |
| --- | --- | --- |
| Participant | Adults above 18y, showing depressive symptoms using objective or subjective criteria using established criteria, e.g. International Statistical Classification of Diseases and Related Health Problems 10th Revision (ICD-10), Diagnostic and Statistical Manual of Mental Disorders, 4th (DSM-IV) and 5th (DSM-V) Edition guidelines) or self-report | No restriction about gender, number of participants, country of origin, comorbidity as long as MDD diagnostic exist |
| Intervention | Combined physical activity and cognitive training:  PA encompasses any bodily movement resulting in energy expenditure, including activities such as cycling, dancing, walking, balance training, yoga, aerobics, or non-aerobic exercises.   Cognitive training involves exercises targeting executive functions like memory, attention, and inhibitory control.  Additionally, dual-tasking or exergaming was considered a combined intervention, provided it aligned with the previously mentioned definitions of PA and cognitive training | No special restriction was made about the PA or cognitive interventions, duration or intensity  Cognitive behavioral therapy CBT, health and education sessions, classes targeted changing way of thinking, and motivational courses were excluded |
| Comparison | The comparison group should have gotten either PA training alone, or cognitive training alone. treatment as usual or with medications was accepted provided that either modality of training was provided beside that | routine care, wait-list, health class, education class, watching documentaries, watching videos were excluded |
| Outcome | included within-group or between-group pre-post scores on at least one test of depression | no restrictions on the types of depression assessment used |
| Study Design | RCT, clinical trials | case studies descriptive studies  cross sectional studies  systematic review  meta-analysis, reviews were excluded |

Table 2. Excluded Studies

Excluded Studies at Full-Text Screening Stage

| Author (s) | Title | Reason for exclusion |
| --- | --- | --- |
| McDougall Jr et al., (2015) | Memory training plus yoga for older adults | Wrong study design |
| Zhu et al., (2022) | Effect of 3-Month Aerobic Dance on Hippocampal Volume and Cognition in Elderly People With Amnestic Mild Cognitive Impairment: a Randomized Controlled Trial | Wrong study design |
| Lin et al. (2020) | Effects of a Combination of Three-Dimensional Virtual Reality and Hands-on Horticultural Therapy on Institutionalized Older Adults’ Physical and Mental Health: Quasi-Experimental Design | Wrong study design |
| Norouzi et al. (2024) | Combined mindfulness-based stress reduction and physical activity improved psychological factors and sleep quality in patients with MDD: A randomized controlled trial study | Wrong study design |
| Cetin et al., (2022) | Virtual reality and motor control exercises to treat chronic neck pain: a randomized controlled trial | Wrong intervention |
| Hong et al., (2023) | Effects of multimodal cognitive exercise program on cognitive function, bone density, blood lipid, fitness, and depression in old women with mild cognitive impairment | Wrong intervention |
| Meldrum et al., (2015) | Effectiveness of conventional versus virtual reality–based balance exercises in vestibular rehabilitation for unilateral peripheral vestibular loss: results of a randomized controlled trial. | Wrong intervention |
| Rutkowski et al., (2022) | Comparison of two exergame-based motor-cognitive trainings: protocol for a pilot study on cognitive, motor, and psychiatric symptoms in patients with huntington's disease (CARE-MH) | Wrong intervention |
| Thompson et al., (2013) | Exercise Leads to Better Clinical Outcomes in Those Receiving Medication Plus Cognitive Behavioral Therapy for Major Depressive Disorder | Wrong intervention |
| Jeong et al., (2021) | Impacts of dance on cognition, psychological symptoms and quality of life in Parkinson's disease | Wrong control group |
| Jóźwik et al., (2021) | Evaluation of the impact of virtual reality-enhanced cardiac rehabilitation on depressive and anxiety symptoms in patients with coronary artery disease: a randomised controlled trial | Wrong control group |
| Merchant et al., (2021) | Motoric cognitive risk syndrome, physio-cognitive decline syndrome, cognitive frailty and reversibility with dual-task exercise | Wrong control group |
| Ozdogar et al. (2023) | Effect of exergaming in people with restless legs syndrome with multiple sclerosis: A single-blind randomized controlled trial | Wrong control group |
| Santos et al., (2015) | Multidisciplinary rehabilitation program: effects of a multimodal intervention for patients with Alzheimer’s disease and cognitive impairment without dementia | Wrong control group |
| Seo et al., (2023) | Virtual reality exercise program effects on body mass index, depression, exercise fun and exercise immersion in overweight middle-aged women: a randomized controlled trial | Wrong control group |
| Szczepańska-Gieracha et al., (2021) | Immersive virtual reality therapy as a support for cardiac rehabilitation: a pilot randomized-controlled trial | Wrong control group |
| Vieira et al., (2018) | Virtual reality exercise on a home-based phase III cardiac rehabilitation program, effect on executive function, quality of life and depression, anxiety and stress: a randomized controlled trial. | Wrong control group |
| Zhou et al., (2020) | Prevalence of depression and its correlative factors among female adolescents in China during the coronavirus disease 2019 outbreak | Wrong control group |
| Li et al. (2018) | Exergames vs. traditional exercise: investigating the influencing mechanism of platform effect on subthreshold depression among older adults | No full text available |
| Yokoi et al., (2022) | Dual-Task Training Combining Cognitive Tasks and Occupations among Japanese Community-Dwelling Older Adults: A Pilot Study | No full text available |

Note: CG: control group; CT: Cognitive Training; PT: Physical Training; RCT: Randomized Controlled Trial

Table 3. Certainty of Evidence

Summary of Findings and Certainty of Evidence (GRADE) for the Effect of Combined Physical and Cognitive Training Compared to Single Interventions on Depressive Symptoms

**Author(s):**

**Question:** Combined physical and cognitive training compared to physical training or cognitive training alone for Adults with depressive symptoms (predominantly clinical populations with neurological and medical comorbidities)

**Setting:** clinical and community settings

**Bibliography:**

| **Certainty assessment** | | | | | | | **№ of patients** | | **Effect** | | **Certainty** | **Importance** |
| --- | --- | --- | --- | --- | --- | --- | --- | --- | --- | --- | --- | --- |
| **№ of studies** | **Study design** | **Risk of bias** | **Inconsistency** | **Indirectness** | **Imprecision** | **Other considerations** | **Combined physical and cognitive training** | **physical training or cognitive training alone** | **Relative (95% CI)** | **Absolute (95% CI)** |  |  |
| **Depressive symptoms** | | | | | | | | | | | | |
| 16 | randomised trials | not serious | serious^a^ | serious^b^ | not serious | none | 363 | 450 | - | **0**  (0.7 lower to 0.15 lower) | ⨁⨁◯◯ Low^a,b^ | CRITICAL |

**CI:** confidence interval

#### Explanations

a. The certainty of evidence was downgraded for inconsistency due to moderate heterogeneity (I² = 67.3%) and variability in effect direction across studies

b. The certainty of evidence was downgraded for indirectness because the included studies predominantly involved heterogeneous clinical populations with neurological and medical comorbidities rather than primary depressive disorder samples

Table 4. Risk of Bias Assessment

**Risk of Bias 2 Assessment**

| **Study** | **D1** | **D2** | **D3** | **D4** | **D5** | **Overall** | **Analysis** | **Targe of RoB** |
| --- | --- | --- | --- | --- | --- | --- | --- | --- |
| Ayari et al., 2023 | some concerns | low risk | low risk | some concerns | low risk | some concerns | Complete Cases | Effect of Assignment |
| Baek et al., 2024 | some concerns | low risk | low risk | some concerns | low risk | some concerns | Complete Cases | Effect of Assignment |
| Bisbe et al., 2020 | low risk | low risk | low risk | low risk | low risk | low risk | Per Protocol | Effect of Adherence |
| Cano-Manas et al., 2020 | low risk | low risk | low risk | some concerns | low risk | some concerns | Complete Cases | Effect of Assignment |
| Carvalho et al., 2020 | low risk | low risk | some concerns | low risk | low risk | some concerns | Intention-to-treat | Effect of Assignment |
| de Rooij et al., 2021 | low risk | low risk | low risk | low risk | low risk | low risk | Intention-to-treat | Effect of Assignment |
| Ferraz et al., 2018 | low risk | low risk | low risk | low risk | low risk | low risk | Complete Cases | Effect of Assignment |
| Galperin et al., 2023 | low risk | low risk | low risk | low risk | low risk | low risk | Complete Cases | Effect of Assignment |
| Khushnood et al., 2021 | some concerns | low risk | low risk | some concerns | low risk | some concerns | Complete Cases | Effect of Assignment |
| Lin et al., 2020 | low risk | low risk | low risk | low risk | low risk | low risk | Intention-to-treat | Effect of Assignment |
| Ozdogar et al., 2020 | some concerns | low risk | low risk | some concerns | low risk | some concerns | Per Protocol | Effect of Adherence |
| Ozdogar et al., 2022 | some concerns | low risk | low risk | some concerns | low risk | some concerns | Per Protocol | Effect of Adherence |
| Song & Park 2015 | some concerns | low risk | low risk | some concerns | some concerns | some concerns | Intention-to-treat | Effect of Assignment |
| Swinnen et al., 2023 | low risk | some concerns | some concerns | low risk | low risk | some concerns | Intention-to-treat | Effect of Assignment |
| Tollar et al., 2019 | low risk | low risk | low risk | low risk | low risk | low risk | Intention-to-treat | Effect of Assignment |
| Yoon et al., 2013 | some concerns | low risk | low risk | low risk | low risk | some concerns | Complete Cases | Effect of Assignment |

**Note:** Risk of bias assessment

S1. Search strategy

PubMed

Search: **("exercis*" OR physical activity OR "treadmill" OR "physical exertion" OR "swim*" OR "walk*" OR "danc*" OR "jogging" OR running OR "cycl*" OR "bicycl*" OR "hiking" OR "tai chi" OR "tai ji" OR "tai-ji" OR "yoga" OR "qigong" OR "qi gong" OR "sport*" OR "physical training" OR "physical intervention*" OR "physical exercise" OR "strength training" OR "weight" OR "resistance training" OR "resistance exercise" OR "muscle training" OR "balance" OR "aerobic training" OR "aerobic exercise" OR "anaerobic training" OR "anaerobic exercise" OR "cardiac rehabilitation" OR "endurance" OR "muscle training" OR "motor training" OR "motor exercise*" OR "pilates" OR "Feldenkrais" OR "motor activity" OR "cardiac rehabilitation" OR "combined" OR "Multimodal" OR "Multi-modal" OR "Multicomponent" OR "Multi-component" OR "simultaneous*" OR "sequential" OR "dual*" OR "exergam*" OR "video gam*" OR "active video game" OR "Wii" OR "Kinect" OR "Nintendo" OR "Balance board" OR "Computer*" OR "Sony" OR "Dance Dance Revolution" OR "EyeToy" OR "Microsoft" OR "XBOX360" OR "Motion Sensing Game" OR "Interaction game") AND("cognitive intervention*" OR "cognitive training" OR "cognitive exercise*" OR "cognitive treatment" OR "cognitive therapy" OR "cognitive class" OR "cognitive stimulat*" OR "cognitive remediation" OR "brain training" OR "brain exercise" OR "brain intervention" OR "brain treatment" OR "attention" OR "executive function*" OR "reasoning" OR "updating" OR "shifting" OR "switching" OR "memory" OR "mental training"OR"neurocognitive" OR "visual-spatial" OR "language" OR "orientation" OR "processing" OR "inhibitory control" OR "inhibition" OR videogam* OR "exergam*" OR "Span Forward" OR "Letter-Number Sequencing" OR "n-back" OR "computer game*" OR "interactive video*" OR "virtual reality" OR "neurocogniti*" OR "neuropsychol*" OR "computerized training") AND ("depressive" OR "depressio*" OR "MDD" OR "dysthymi*" OR "mood disorder" OR "affective disorder") NOT ("child*" OR "animal*" OR "mice" OR "rats" OR "rat" OR "fish" OR "monkey" OR cell OR "youth*")** Filters: **Clinical Trial, Randomized Controlled Trial, English**

**Web of Science**

**("exercis*" OR physical activity OR "treadmill" OR "physical exertion" OR "swim*" OR "walk*" OR "danc*" OR "jogging" OR running OR "cycl*" OR "bicycl*" OR "hiking" OR "tai chi" OR "tai ji" OR "tai-ji" OR "yoga" OR "qigong" OR "qi gong" OR "sport*" OR "physical training" OR "physical intervention*" OR "physical exercise" OR "strength training" OR "resistance" OR "muscle training" OR "balance" OR "aerobic " OR "aerobic exercise" OR "anaerobic " OR "cardiac rehabilitation" OR "endurance" OR "muscle training" OR “motor training” OR “motor exercise*” OR “active video game” OR "Wii" OR "Kinect" OR "pilates" OR "Feldenkrais" OR "motor activity" OR "Combined" OR "Multimodal" OR OR "simultaneous*" OR "sequential" OR "dual*")**

**AND**

**("cognitive intervention*" OR "cognitive training" OR "cognitive exercise*" OR "cognitive stimulat*" OR "cognitive remediation" OR "brain training" OR "brain exercise" OR "brain intervention" OR OR "attention" OR "executive function*" OR "reasoning" OR "updating" OR "shifting" OR "switching" OR "memory" OR "mental training" OR "neurocognitive" OR "visual-spatial" OR "language" OR "orientation" OR "processing" OR "inhibitory control" OR "inhibition" OR videogam* OR "exergam*" OR "Span Forward" OR "Letter-Number Sequencing" OR "n-back" OR "computer game*" OR "interactive video*" OR "virtual reality" OR "neurocogniti*" OR "neuropsychol*" OR "computerized training")**

**AND**

**("depressive” OR “depressio*" OR "MDD" OR "dysthymi*" OR "mood disorder" OR "affective disorder" OR "bipolar") NOT**

**(“child*” OR “animal*” OR “mice” OR "rats" OR "rat" OR "fish" OR cell OR “youth*”)** (Topic) and **Review Article** or **Book Chapters** or **Editorial Material** or **Meeting** (Exclude – Document Types) and **German** or **Spanish** or **Chinese** or **French** or **Arabic** or **Italian** or **Portuguese** or **Russian** (Exclude – Languages) and **Zoology** or **Acoustics** or **Anthropology** or **Audiology Speech Language Pathology** or **Biotechnology Applied Microbiology** or **Business Economics** or **Dentistry Oral Surgery Medicine** or **Emergency Medicine** or **Family Studies** or **Gastroenterology Hepatology** or **Food Science Technology** or **Radiology Nuclear Medicine Medical Imaging** or **Plant Sciences** or **Energy Fuels** or **Anesthesiology** or **Social Sciences Other Topics** or **Medical Informatics** or **Mathematics** or **Mathematical Computational Biology** or **Geochemistry Geophysics** or **Biomedical Social Sciences** or **Materials Science** or **Physics** or **Geriatrics Gerontology** or **Chemistry** or **Pharmacology Pharmacy** or **Engineering** or **Veterinary Sciences** or **Virology** or **Water Resources** or **Cell Biology** or **Urology Nephrology** or **Electrochemistry** or **Biophysics** or **Astronomy Astrophysics** or **Imaging Science Photographic Technology** or **Metallurgy Metallurgical Engineering** or **Mineralogy** or **Marine Freshwater Biology** or **Information Science Library Science** or **Immunology** or **History Philosophy Of Science** or **Hematology** or **Geography** or **Genetics Heredity** or **Instruments Instrumentation** or **Computer Science** or **Nursing** or **Agriculture** or **Respiratory System** or **Oncology** or **Integrative Complementary Medicine** or **Surgery** or **Biochemistry Molecular Biology** or **Toxicology** or **Telecommunications** or **Social Issues** or **Remote Sensing** or **Polymer Science** or **Pediatrics** or **Optics** or **Oceanography** or **Nuclear Science Technology** or **Microbiology** or **Meteorology Atmospheric Sciences** or **Religion** or **Robotics** or **Spectroscopy** or **Substance Abuse** (Exclude – Research Areas) and **Environmental Sciences** or **Environmental Studies** or **Geosciences Multidisciplinary** or **Nutrition Dietetics** or **Ecology** or **Neuroimaging** or **Ophthalmology** or **Otorhinolaryngology** or **Paleontology** or **Geology** (Exclude – Web of Science Categories)

Scopus

("depressive” OR “depressio*" OR "MDD" OR "dysthymi*" OR "mood disorder" OR "affective disorder" OR "bipolar")

AND

("Combined" OR "Multimodal" OR "Multi-modal" OR "Multicomponent" OR "Multi-component" OR "simultaneous*" OR "sequential" OR "dual*")

AND

("cognitive intervention*" OR "cognitive training" OR "cognitive exercise*" OR "cognitive treatment" OR "cognitive therapy" OR "cognitive class" OR "cognitive stimulat*" OR "cognitive remediation" OR "brain training" OR "brain exercise" OR "brain intervention" OR "brain treatment" OR "attention" OR "executive function*" OR "reasoning" OR "updating" OR "shifting" OR "switching" OR "memory" OR "mental training" OR "neurocognitive" OR "visual-spatial" OR "language" OR "orientation" OR "processing" OR "inhibitory control" OR "inhibition" OR videogam* OR "exergam*" OR "Span Forward" OR "Letter-Number Sequencing" OR "n-back" OR "computer game*" OR "interactive video*" OR "virtual reality" OR "neurocogniti*" OR "neuropsychol*" OR "computerized training")

AND

("exercis*" OR physical activity OR "treadmill" OR "physical exertion" OR "swim*" OR "walk*" OR "danc*" OR "jogging" OR running OR "cycl*" OR "bicycl*" OR "hiking" OR "tai chi" OR "tai ji" OR "tai-ji" OR "yoga" OR "qigong" OR "qi gong" OR "sport*" OR "physical training" OR "physical intervention*" OR "physical exercise" OR "strength training" OR "weight" OR "resistance training" OR "resistance exercise" OR "muscle training" OR "balance" OR "aerobic training" OR "aerobic exercise" OR "anaerobic training" OR "anaerobic exercise" OR "cardiac rehabilitation" OR "endurance" OR "muscle training" OR “motor training” OR “motor exercise*” OR “exergam” OR “active video game” OR "Wii" OR "Kinect" OR "pilates" OR "Feldenkrais" OR "motor activity" OR "cardiac rehabilitation")

NOT (“child*” OR “animal*” OR “mice” OR "rats" OR "mouse" OR "rat" OR "fish" OR cell OR “youth*”)

Figure 1. Forest Plot of the effect sizes restricted to low risk of bias studies only (Sensitivity Analyses).

*Note*. An effect size below zero favours the combined PT and CT interventions over PT alone. Abbreviations: EG: Experimental Group; CG: Control Group; SMD: Standardized Mean Difference; CI: Confidence Interval
